# Enhanced power factor by transition metal substitution in the thermoelectric Kagome metal Ni_3_Sn

**DOI:** 10.1039/d6ta01928e

**Published:** 2026-09-25

**Authors:** Kim-Isabelle Mehnert-Birk, Saff E. Awal Akhtar, Blair F. Kennedy, Ankit Kumar, Neophytos Neophytou, Jan-Willem G. Bos

**Affiliations:** a EaStCHEM School of Chemistry, University of St Andrews North Haugh, St Andrews KY16 9ST UK j.w.g.bos@st-andrews.ac.uk; b School of Engineering, University of Warwick Coventry CV4 7AL UK N.Neophytou@warwick.ac.uk

## Abstract

Metallic thermoelectric materials offer promise in terms of elemental abundance and avoiding rare or toxic elements. Here, we demonstrate that substitution of Co in the Kagome metal Ni_3_Sn leads to an enhanced Seebeck effect (*S*_max_ = −55 µV K^−1^), which supports a promising thermoelectric power factor, PF = 5 mW m^−1^ K^−2^ and figure of merit, *zT* = 0.1. Density Functional Theory calculations show that the metallic electronic structure consists of dispersive bands around the Fermi level (*E*_F_) and a combination of flattened out and dispersive bands below it. Boltzmann transport equation modelling shows that the resulting step-change increase in density of states (DOS) from the flattened bands, leads to the large observed *S* values through selective carrier scattering. With Co substitution, the alignment of *E*_F_ to the high DOS region is improved, enhancing *S*. Substitution with Cu moves *E*_F_ away from the high DOS region, reducing *S*. This work shows that the behaviour of Ni_3_Sn can be understood by DOS scattering driven carrier filtering. As also proposed by Mott, this is a robust effect that occurs in degenerate metallic conditions, leading to more than two times enhancement in *S* and applies to many more intermetallic materials with an appropriately tuned band structure.

## Abstract 2

Co-substitution in Ni_3_Sn brings large density of states regions closer to the Fermi level, enabling energy filtering through selective scattering of low-energy carriers. This increases the thermoelectric power factor.

## Graphical abstract

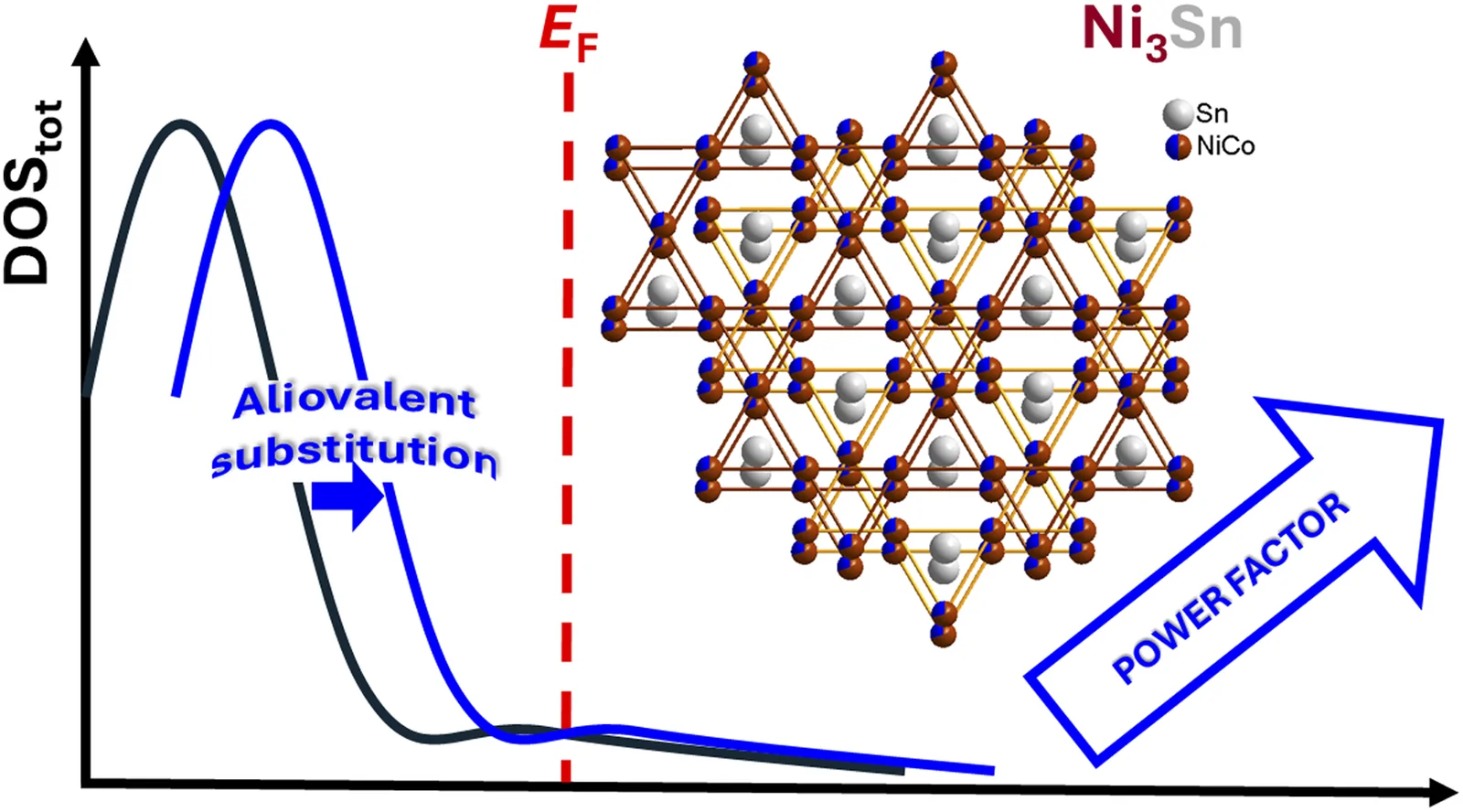

## 1. Introduction

Thermoelectrics are dominated by low bandgap semiconductor materials, often based on alloys of Bi, Pb and Te,^1–3^ while metals are typically excluded because of their low Seebeck coefficient (*S*) and therefore low power factor, PF = *S*^2^*σ*, while also having high thermal conductivity (*κ*).^4^ Recent developments, however, have demonstrated that a large energy variation in the conductivity (*σ*) of certain metals can be achieved if the Fermi level (*E*_F_) is placed near regions of flat bands with high density of states (DOS).^5^ Here, the flat bands scatter dispersive electron-like band carriers and effectively implement an energy filtering mechanism for low energy carriers. This removes transport compensation between hole- and electron-like carriers and increases *S* and the PF. For ideal metals, whose thermal conductivity is dominated by electronic transport, *i.e. κ* ∼ *κ*_el_ = *LσT*, the thermoelectric figure of merit, *zT* = (PF/*κ*)*T* reduces to *zT* = *S*^2^/*L*, which only depends on *S* and the Lorenz number (*L*). Thus, in special cases, metal alloys can provide simultaneously high *S* and PF at room temperature, and high *zT*, making them ideal for active cooling applications.^6,7^

Selective carrier scattering has recently been successfully used to design metallic alloys, including Ni–Au with ultra-high PF = 34 mW m^−1^ K^−2^.^5^ To understand this, it is necessary to consider the Transport Distribution Function (TDF): *G*(*E*) = *τ*(*E*)*v*^2^(*E*)*g*(*E*) where, *τ*, *ν* and *g* are the energy dependent scattering time, band structure velocity, and density of states, respectively. *σ* is proportional to *G*(*E*), however, *S* is obtained by integrating *G*(*E*) × (*E* − *E*_F_), which is negative below *E*_F_ and positive above.^8–12^ Hence contributions to *S* from above and below *E*_F_ cancel out if *G*(*E*) does not vary much around *E*_F_, which is the situation for regular metals such as Cu and Al. However, as shown by Mott, metals combining localised (*e.g.* 3d, 4f) and dispersive bands, can show such a step change in *G*(*E*), which is largely caused by the change in carrier lifetime *τ* ∝ *g*(*E*)^−1^ caused by a change in *g*(*E*).^13,14^ If this occurs near *E*_F_ (and only on one side, *e.g.* in the lower energy region) it removes the compensation between hole- and electron-like carriers, increasing *S*. This can be directly seen from Mott's formula:1
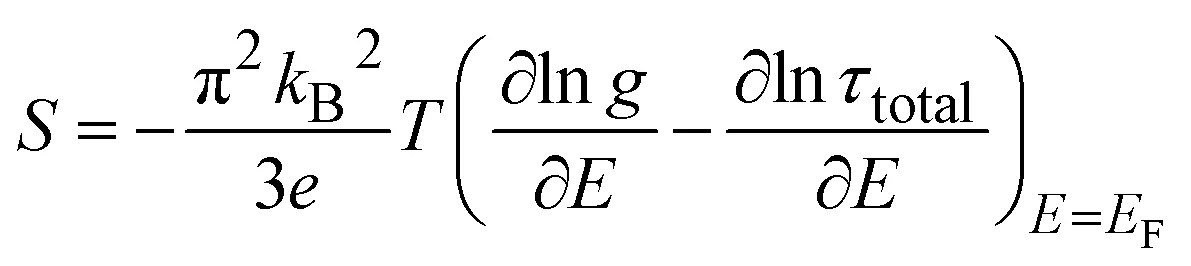
Here, the energy derivative of *τ*(*E*) at *E*_F_ appears explicitly as a contributing factor to *S*, in addition to the energy dependence of *g*(*E*) that dominates in semiconductors. Selective scattering has been used to explain the large *S* for the Ni–Au alloys and of regular thermocouple alloys.^4,5^ Compared to constantan (Ni–Cu; PF ≤ 10 mW m^−1^ K^−2^),^15^ Ni–Au has a greater step in *g*(*E*) due its larger lattice parameter, which flattens the Ni 3d-band below *E*_F_ due to weaker orbital overlap, increasing *g*(*E*) and scattering rates of states below *E*_F_.

Step changes in *g*(*E*) can also be found in intermetallic compounds with ordered structures of 3d metals and main group atoms. These materials offer advantages from a sustainability perspective if scarce and/or toxic elements can be avoided.^16^ In a previous work we have discussed a method to theoretically examine electronic transport in such compounds allowing high PFs.^17^ The discovered favourable parameters are (i) a high degree of transport asymmetry between the bands around *E*_F_ (*e.g.* a mix of flat and dispersive bands), (ii) strong inter-band scattering and (iii) a large degree of band overlap.

Intermetallic compounds that have been explored recently include cubic Ni_3_Ge (PF = 8 mW m^−1^ K^−2^)^18^ and the Kagome metal Ni_3_Sn_1−*x*_In_*x*_ (PF = 4 mW m^−1^ K^−2^).^19^ Kagome materials in particular are of great interest as they contain topologically frustrated chemical bonds, which can lead to a spike in *g*(*E*) due to a near zero band dispersion.^20–23^ This spike in *g*(*E*), strongly suppresses the *τ*(*E*) of overlapping dispersive bands through inter-band scattering, resulting in strong asymmetry in *G*(*E*) and thereby high *S*. For Ni_3_Sn, *S* = −22 µV K^−1^ was measured at 300 K, increasing to *S* = −45 µV K^−1^ for 50% substituted Ni_3_Sn_0.5_In_0.5_, with both compositions tending towards *S* = −50 µV K^−1^ at high temperatures.^19^ In that work, In substitution removes electrons from the system, moving *E*_F_ closer to the step change of *g*(*E*).

In the present work, transition metal substitution on the Ni position was used to tune *E*_F_. To this end, a series of Ni_3−*x*_M_*x*_Sn compounds with M = Co and Cu (*x* ≤ 0.2) were prepared and investigated experimentally and theoretically. Here, M = Co, Cu substitute directly in the Kagome layers and will change the position of *E*_F_ with respect to the high *g*(*E*) band itself. This approach affords both hole (Co) and electron doping (Cu) and for the first time allows the impact of both types of doping to be assessed. Co substitution strongly enhances *S* to a maximum of −50 µV K^−1^ at 350 K, comparable to the Ni_3_Sn_1−*x*_In_*x*_ system. Cu substitution leads to a small reduction in *S*. A larger ingot of the best composition Ni_2.9_Co_0.1_Sn was prepared to assess the impact of texturing and yielded *zT* = 0.05–0.06 at 350 K with further moderate increase to *zT* = 0.08–0.1 at 800 K.

Our work shows that a minimal two-band model using an overlapping electron-like dispersive (light) and hole-like flattened out (heavy) band is sufficient to capture the experimental trends and reproduce the experimental *S*(*T*) in near-quantitative manner. This model shows that the key parameters for strong enhancement of *S* are band alignment and inter-band scattering. For practical applications, *S* needs to exceed 100 µV K^−1^. Although our results are not there yet, this work shows that this can be achieved by engineering the position of *E*_F_ and the steepness of the scattering DOS edge. The success of the minimal model based on a limited parameter set confirms that the *S* and PF enhancement is based on a robust mechanism and does not rely on special electronic structures or unique position of *E*_F_ that might be missed in a real sample. Like the bandgap in semiconductors, band alignment and selective carrier scattering in metals is therefore another reliable route to achieve large *S* and thermoelectric performance.

## 2. Results

### 2.1 Structure evolution

An initial set of Ni_3−*x*_M_*x*_Sn compounds with M = Co and Cu (*x* ≤ 0.2) were prepared on a 5 gram scale to explore the phase behaviour and solubility of the metals. This initial exploration revealed moderate texturing of the hot-pressed discs, and that the M = Co, *x* = 0.1 sample had the most promising thermoelectric performance. To be able to measure the thermoelectric properties parallel and perpendicular to the pressing direction, a taller ingot of the M = Co, *x* = 0.1 composition was prepared. Fig. 1c shows the directions along which thermoelectric and XRD data were collected. The samples are stable for at least 24 months.

**Fig. 1:**
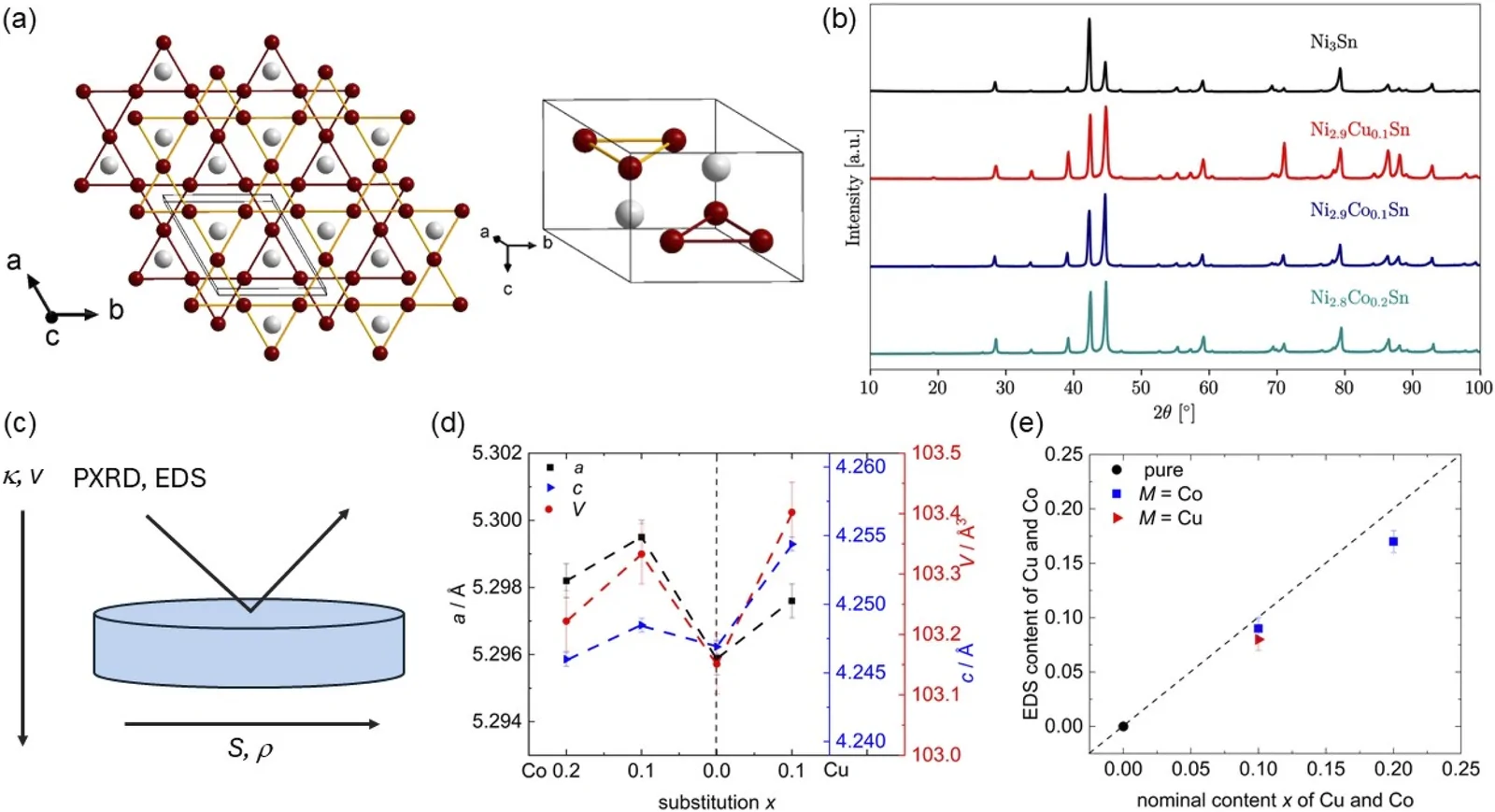
(a) Layered Kagome structure of Ni_3_Sn with Ni in red and Sn in grey. (b) XRD patterns for Ni_3−*x*_M_*x*_Sn samples collected from hot-pressed discs. (c) Illustration of XRD and property measurement directions. (d) Refined lattice parameters and unit cell volume (*a*, *c* and *V*) for the Ni_3−*x*_M_*x*_Sn samples. (e) Comparison of nominal and EDS content for the Co/Cu substitution series.

Ni_3_Sn has a hexagonal *P*6_3_/*mmc* structure, first reported 1937,^24^ with Ni Kagome layers that are offset by (½, ½, 0) and stacked in AB sequence. This leads to Sn being coordinated in an anti-cuboctahedron. The Kagome layer and unit cell are illustrated in Fig. 1a. There are several conflicting reports on the high temperature (>700 °C) structure of Ni_3_Sn. Some state that the structure remains *P*6_3_/*mmc*,^25^ others report an orthorhombic structure,^26^ and still others suggest a cubic BiF_3_-type structure^27–29^ or the cubic L1_2_-ordered Cu_3_Au structure, in which Ni_3_Ge crystallises.^18^ In this work, we have only found the hexagonal Kagome structure, both from arc melting and quenching after annealing at 1050 °C. Our property measurements stay below 700 °C up to which the hexagonal *P*6_3_/*mmc* structure is stable.^29^ Additionally, no phase transition was observed in our heat capacity measurements (shown in the SI), in agreement with the stability of the *P*6_3_/*mmc* structure. The hexagonal *P*6_3_/*mmc* symmetry was confirmed using powder X-ray diffraction (XRD, Fig. 1b and S1, S2 in the SI). The M = Co, *x* = 0.2 sample has a small impurity reflection at 2*θ* = 27.2°, suggesting that the solubility limit has been reached, which is confirmed by EDS analysis (discussed below), suggesting an actual value *x* ∼0.17. Sample M = Cu, *x* = 0.2 showed poor crystallinity and has been omitted from this manuscript. XRD data collected after property measurements confirmed an unchanged structure (Fig. S3).

Rietveld analysis was performed to extract lattice parameters (Table S1 and Fig. 1d) and to quantify the texturing that is evident from the XRD datasets. Visual inspection of the three main reflections show that the parent material Ni_3_Sn has a much stronger (0 0 2) reflection (at 42.5°) than the Co and Cu substituted samples, which all have similar intensity ratios. This effect is consistent over multiple batches of hot-pressed discs and is also present in data collected on powdered discs (Fig. S1). Quantitative analysis using the March–Dollase model (MD) with unique (0 0 2) axis illustrates this difference with near identical values of 0.72–0.74 for the Co/Cu substituted samples and 0.59 for Ni_3_Sn (Table S2). These values are indicative of moderate texturing (a value of 1 means the absence of texturing). The larger Ni_2.9_Co_0.1_Sn ingot has almost no texturing with MD values 0.9–1.0, suggesting sensitivity to sample size during hot-pressing (Fig. S2). Additionally, allowing for anisotropic peak broadening with respect to the (0 0 2) texturing axis was found to improve the fit. The refined axial and equatorial microstrain parameters are summarised in Table S2. This reveals similar values with the equatorial strain (∼0.6%) around 50% larger than the axial values (∼0.4%). Notably, as with the texturing index, the larger Ni_2.9_Co_0.1_Sn ingot has much more similar axial and equatorial microstrain with values of 0.46% and 0.38%, respectively. The cause of the microstrain broadening within the Kagome layers is not clear at present. Both texturing and microstrain are affected by changes in processing, which for these samples include the use of a 4-arc instead of a regular single-arc furnace, and multiple hot-press runs for the larger sample instead of a single consolidation step (see Methods for details).

The lattice parameters (Fig. 1d) do not change very much upon substitution with Co/Cu, staying within 0.003–0.004 Å of the Ni_3_Sn parent material. With such small differences the lattice parameters become very sensitive to systematic errors. The relaxed lattice parameters from DFT calculations predict that Co/Cu substitution should both lead to an increased cell volume *V*. This trend is indeed observed in the data, but caution is warranted as only small amounts of 3.3% (*x* = 0.1) and 6.7% (*x* = 0.2) of Ni are being replaced, and the experimental changes are small. Analysis of the chemical bonding shows that this can be understood as a mixture of localised charge on the transition metals and delocalised electrons, originating from Sn (see SI for full details). In the parent material Ni has a positive charge density and Sn a negative one, showing charge accumulation and depletion respectively. In the substituted materials, Cu has a negative charge density (like Sn), leading to reduced interaction with the delocalised charge (Fig. S15). This leads to increased bond distances and unit cell volume. By contrast, Co has a positive charge density, like Ni but higher, leading to increased interaction with the delocalised charge and shorter Co–Sn bond distances. However, this charge transfer to Co leads to increased Ni–Sn bond distances and a net expansion in unit cell volume. So for both substitutions the unit cell volume increases, despite their different affinity toward valence charge density.

Scanning electron microscopy (SEM) imaging and energy dispersive X-ray spectroscopy (EDS) analysis confirmed well-sintered microstructures, with some larger macropores, for all samples, and the homogenous distribution of all elements, including the dopants (Fig. S4–S7). The amount of Co/Cu found in the samples, obtained from integrating large area maps, is reported in Fig. 1e and Table S3. This shows that the Co/Cu uptake for the samples is close to the nominal values, in agreement with the absence of major impurities in the XRD patterns. For the M = Co, *x* = 0.2 sample, the EDS composition (*x* ∼0.17) is slightly lower than the nominal value, in keeping with the observation of a small impurity peak in XRD for that sample. The gravimetric densities (listed in Table S4) of the samples fall between 88–100% compared to theoretical (crystallographic) densities, which is linked to the presence of larger pores shown in the SEM imaging (Fig. S4–S7), which do not affect thermoelectric properties strongly with the materials generally well sintered.

Overall, the structural analysis confirms that the smaller ingots from the initial series have a stronger degree of texturing, with a significant difference between Ni_3_Sn and the Co/Cu substituted samples. This is less pronounced for the larger ingot, which is nearly isotropic, both in terms of texturing and anisotropic microstrain. SEM and EDS confirm dense microstructures, chemical homogeneity and compositions of the samples with successful substitution of Co/Cu at near expected levels.

### 2.2 Thermoelectric measurements

Temperature dependent *S*(*T*), electrical resistivity *ρ*(*T*) = 1/*σ*(*T*), PF(*T*), weighted mobility *µ*_w_(*T*) calculated from *S* and *ρ*,^30^*κ*(*T*) and *zT* are presented in Fig. 2. The measured data is shown by the symbols, whereas Boltzmann transport calculations with a two-band model are shown by solid lines. The compositional dependence at 350 K and 800 K is shown in Fig. S8 and *S* and *ρ* cycling data is shown in Fig. S9–S12 in the SI. Here, 
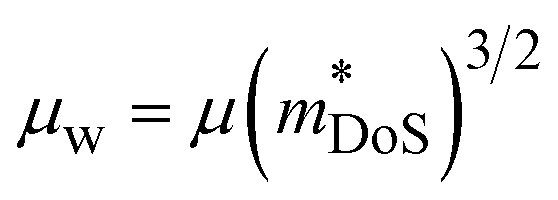
 is used as a proxy for the mobility (*µ*) due to the difficulty in obtaining Hall data on these highly metallic samples.^30^ This expression, valid for a parabolic band, weights *µ* by the density of states 
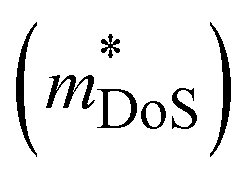
 and is a measure of the electronic quality of a thermoelectric material, which provides useful additional information in addition to the PF, which is dependent on carrier concentration.

**Fig. 2:**
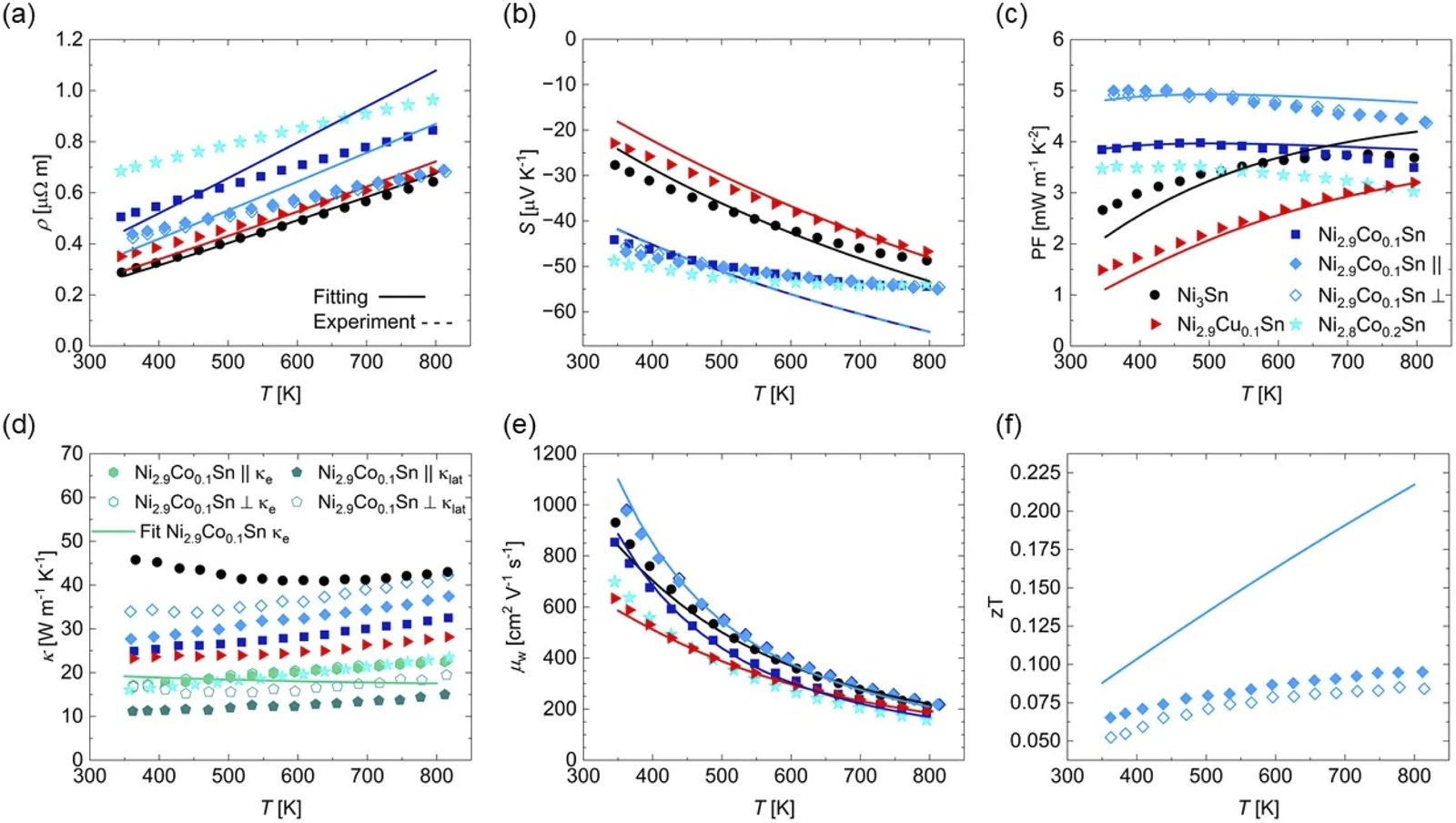
Measured TE coefficients (symbols) for all systems examined, pristine (Ni_3_Sn-black), a system with 3.3% Cu substitution (Ni_2.9_Cu_0.1_Sn – red), a system with 3.3% Co substitution (Ni_2.9_Cu_0.1_Sn – navy blue) and an additional case of 6.67% Co substitution (Ni_2.8_Co_0.2_Sn – cyan). Additionally, measurements for the large Ni_2.9_Co_0.1_Sn ingot (parallel and perpendicular to the hot-press direction for a larger sample are shown (blue). (a) Seebeck coefficient *S*, (b) electrical resistivity *ρ*, (c) power factor PF, (d) thermal conductivity *κ* (including *κ*_el_ and *κ*_lat_ for the large Ni_2.9_Co_0.1_Sn ingot), (e) weighted mobility *µ*_w_ and (f) figure of merit *zT*. Solid lines are fits using the parabolic two-band model. The solid line in panel (f) is calculated using *zT* = *S_2_*/*L*.

All samples have an increasing *S*(*T*) in absolute terms. The Ni_3_Sn parent material has *S* = −28 µV K^−1^ at 350 K, increasing to −49 µV K^−1^ at 800 K, which is comparable to the Ni_3_Sn sample reported previously.^19^ The impact of Co/Cu substitution is most pronounced near RT. Cobalt substitution leads to a significant increase to −44 µV K^−1^ (*x* = 0.1) and −49 µV K^−1^ (*x* = 0.2). By contrast, Cu substitution is detrimental and yields *S* = −22 µV K^−1^ at 350 K (*x* = 0.1). Hence removing electrons and lowering *E*_F_ leads to enhanced *S*, which is consistent with the results for Ni_3_Sn_1−*x*_In_*x*_.^19^ With increasing temperature, the difference in *S*(*T*) reduces with the Co samples achieving the highest *S* = −55 µV K^−1^ at 800 K and the Cu sample reaching −46 µV K^−1^. The *S*(*T*) for the larger Ni_2.9_Co_0.1_Sn ingot is identical to the sample from the initial series, demonstrating the robustness of the increase in *S*(*T*) with Co substitution. As expected from the absence of texturing from XRD, the *S*(*T*) parallel and perpendicular to the hot-press direction are identical.

All samples have metallic *ρ*(*T*) with Ni_3_Sn showing the lowest values. Both measurement directions for the large ingot have identical *ρ*(*T*) as expected in the absence of texturing, but with a 20% lower magnitude compared to the initial series. This reduction in *ρ*(*T*) is attributed to improved homogeneity and density, linked to its preparation using tetra arc melting and sequential hot-pressing steps to obtain a fully dense sample.

In the initial series, the parent material Ni_3_Sn has the highest weighted mobility *µ*_w_, followed by the Co substituted samples and then the Cu substituted samples. However, the large Ni_2.9_Co_0.1_Sn ingot achieves slightly larger *µ*_w_ than the parent material, which is because of its lower *ρ*(*T*), reflecting an improved *µ* due to its higher crystallinity linked to processing. Across all samples, the difference in *µ*_w_(*T*) is largest near room temperature, and approaches similar values at higher temperatures.

Finally, in the initial series the 350 K PF increases from 2.6 mW m^−1^ K^−2^ for Ni_3_Sn to a peak value of 3.9 mW m^−1^ K^−2^ for the *x* = 0.1 Co sample. The large Ni_2.9_Co_0.1_Sn ingot achieves even higher PF of 5.0 mW m^−1^ K^−2^ at 350 K, decreasing slightly to 4.4 mW m^−1^ K^−2^ at 800 K, reflecting its higher crystallinity. At high temperatures, the Co and unsubstituted samples in the initial series achieve similar peak PF = 3.5 mW m^−1^ K^−2^. The Cu substituted sample, which has a low PF = 1.5 mW m^−1^ K^−2^ at 350 K, starts to approach the other samples at high temperatures with PF = 3.2 mW m^−1^ K^−2^. The experimental data show that the impact of element substitution is diminished as temperature increases, which will be explored further below when modelling the properties of these materials.

Fig. 2e shows the *κ*(*T*) of the Ni_3−*x*_M_*x*_Sn compositions calculated with thermal diffusivity *α* from LFA measurements, heat capacity *C*_p_ from DSC measurements (see Fig. S13) and density *ρ*_d_ from physical dimensions. The *κ*(*T*) for Ni_3_Sn is notably different to that of the substituted samples with both a larger magnitude and decreasing temperature dependence, which is typical for phonon dominated heat transport. By contrast, the substituted samples have a lower linear *κ*(*T*), suggesting that the phonon part is suppressed through defect scattering. A similar trend is observed in the Ni_3_Sn_1−*x*_In_*x*_ system.^19^ Additionally, the fact that *κ*(*T*) is increasing suggests that *κ*_el_ = *LσT* is now the dominant contribution, as the increase in *T* would compensate the decrease of *σ*. Due to the discussed texturing observed in XRD and measurements of TE properties in different directions, only for the large Ni_2.9_Co_0.1_Sn ingot a full set of data is available, which enables determination of *κ*_lat_ = *κ* − *κ*_el_ in both measurement directions and calculation of figure of merit *zT*. A two-band model, discussed below, was used to calculate the Lorenz number *L*. This yielded *L* ≈ 1.9 × 10^−8^ W Ω K^−2^ (see Fig. S20 in the SI), which is notably lower than the free electron value (*L* = 2.44 × 10^−8^ W Ω K^−2^). The measured total *κ*(*T*) for the large ingot is similar to the *x* = 0.1 Co sample from the initial series. However, 5–6 W m^−1^ K^−1^ higher values are found for the perpendicular direction, which may reflect the larger experimental uncertainty for that dataset, which was collected on a smaller 3 × 3 mm^2^ cut-out of the ingot. The calculated *κ*_el_(*T*) and extracted *κ*_lat_(*T*) are shown in Fig. 2d. This reveals large *κ*_el_ = 17 W m^−1^ K^−1^ at 350 K, increasing to 23 W m^−1^ K^−1^ at 800 K, which drives the overall increase in *κ*(*T*). Notably, the *κ*_el_(*T*) from the parabolic two-band model fits well with the experimentally obtained data. The resulting *κ*_lat_(*T*) falls between 11 W m^−1^ K^−1^ (‖) and 17 W m^−1^ K^−1^ (⊥) at 350 K, increasing to 15 W m^−1^ K^−1^ (‖) and 20 W m^−1^ K^−1^ (⊥) at 850 K. This does not follow the usual decrease due to increasing Umklapp phonon scattering, but instead follows the strong increase in *C*_p_ (Fig. S13) due to the combined electronic and thermal expansion contributions. Use of *C*_p_ from the Dulong Petit approximation recovers a decreasing *κ*_lat_(*T*). The experimental *κ*_lat_(*T*) agrees well with an estimate based on the mean velocity of sound (*v*_m_) using:^31,32^2
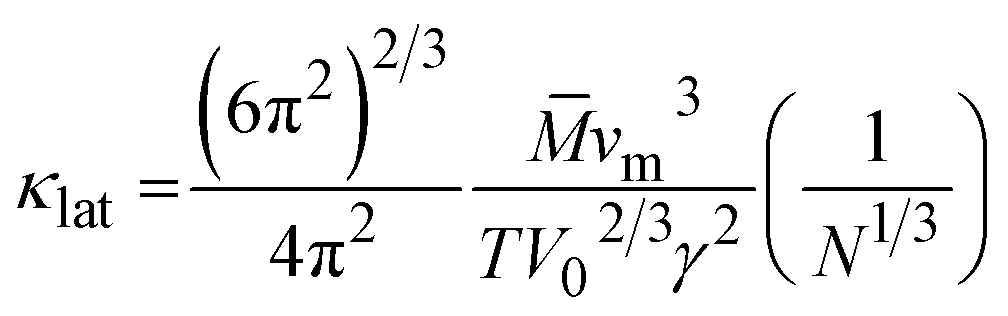
where *M̄* is the average mass per atom, *V*_0_ the volume per atoms and *N* is the number of atoms in the primitive unit cell. Velocity of sound measurements and calculated elastic moduli are summarised in Table S5. Substituting the relevant values in eqn (2) yields *κ*_lat_ = 10 W m^−1^ K^−1^ at 350 K in good agreement with the measured data.

Finally, Fig. 2f shows the calculated *zT* = (*S*^2^*/ρ*κ)*T* values for the large Ni_2.9_Co_0.1_Sn ingot, which increase from 0.065 (‖) and 0.052 (⊥) at 350 K to 0.095 (‖) and 0.085 (⊥) at 800 K. Although not explicitly reported in that study, this is similar to the Ni_3_Sn_1−*x*_In_*x*_ system^19^ and constantan alloys (*zT* ∼0.15)^15^, but below the highest *zT* ∼0.5 for metallic thermoelectrics found in the Ni_1−*x*_Au_*x*_ alloys.^5^ The two-band parabolic band model predicts a highest *zT* = 0.2 at 800 K for an ideal metallic thermoelectric, *i.e.* using *zT* = *S_2_*/*L* with *S* and *L* values from the two-band model. This is around double the actual *zT*, in line with the comparable *κ*_lat_ and *κ*_el_ contributions in this material (Fig. 2d).

### 2.3 Electronic structure calculations

To assess the impact of the Co/Cu substitution, supercell DFT calculations were undertaken, and these were used to inform a two-band transport model. Fig. 3 shows the GGA-PBE calculated band structures of pristine Ni_24_Sn_8_ and its substituted counterparts, Ni_23_CuSn_8_ and Ni_23_CoSn_8_. Experimentally, the Cu and Co concentrations in Ni_3_Sn are approximately 3.3% (*x* = 0.1) and 6.7% (*x* = 0.2, Co only). For theoretical calculations a 2 × 2 × 1 pristine supercell (Ni_24_Sn_8_), with ∼4.2% Co and Cu substitution was chosen as the intermediate experimental substitution range (3.3–6.7%). This provides a realistic and computationally feasible representation of the substituted systems. The calculated band structures of the parent materials and doped compositions show several bands crossing *E*_F_, indicating metallic character with notably flattened regions (at *E* ∼ −0.2 eV) just below *E*_F_ = 0 eV in each case.

**Fig. 3:**
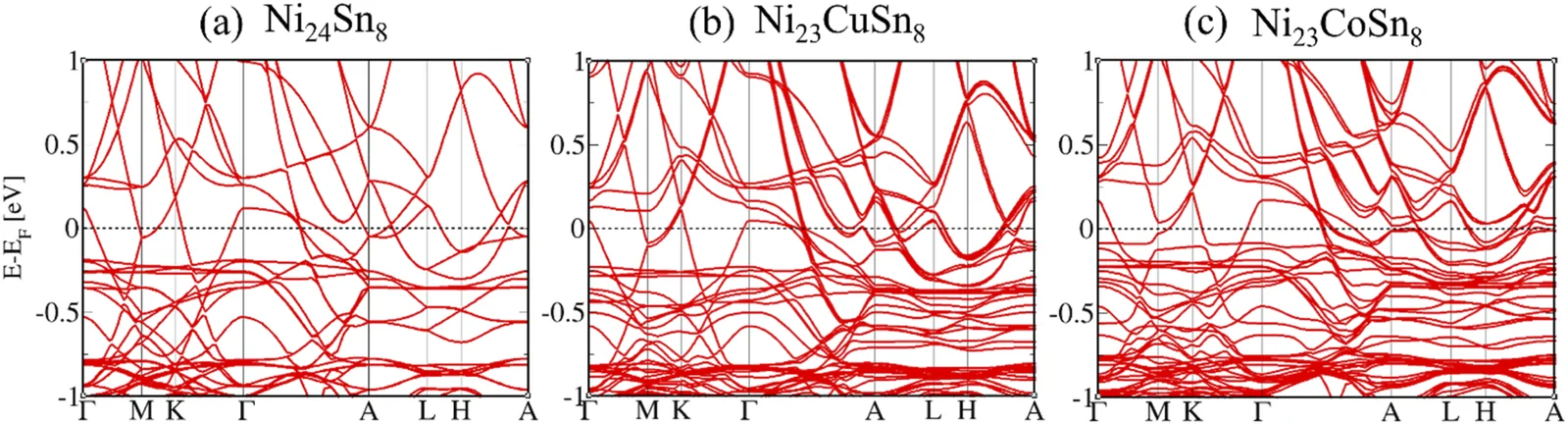
Electronic band structures calculated with GGA-PBE of the (a) parent material – Ni_24_Sn_8_, (b) 4.2% Cu substituted compound – Ni_23_CuSn_8_ and (c) 4.2% Co substituted compound – Ni_23_CoSn_8_.

Fig. 4a better illustrates the implications of the flattening band regions by showing the DOS for the three materials. In all cases, the DOS consists of a low DOS region at high energies above *E*_F_ and a large DOS region for energies below *E*_F_. As shown in the SI (Fig. S14), the large DOS increases are attributed to the d-orbitals of Ni. In fact, Ni-d orbitals strongly dominate the DOS both below and above *E*_F_, while the second but smaller contribution above *E*_F_ comes from Sn-p states, hybridized with Ni-d states, Ni(d)-Sn(p). In Fig. 4a, the basis Ni_24_Sn_8_ material is shown by the black line, for which the sharp increase appears below *E*_F_ at approximately −0.21 eV. In the case of the Cu substituted material, the sharp increase (red line) is shifted to even lower energies, around −0.238 eV away from *E*_F_. On the other hand, for the Co substituted material, the sharp increase (blue line) is now only at about −0.177 eV below *E*_F_. A smaller peak appears closer to *E*_F_ in the Co case, before the large d-orbital peak. This makes the effective shift for the Co material somewhat larger. This shift of the sharp DOS increase around *E*_F_ is the reason for the large *S* variations as described below. Note that whilst the calculations place *E*_F_ at zero by definition, from a chemical perspective these changes correspond to insertion (Cu) and removal of charge carriers (Co).

**Fig. 4:**
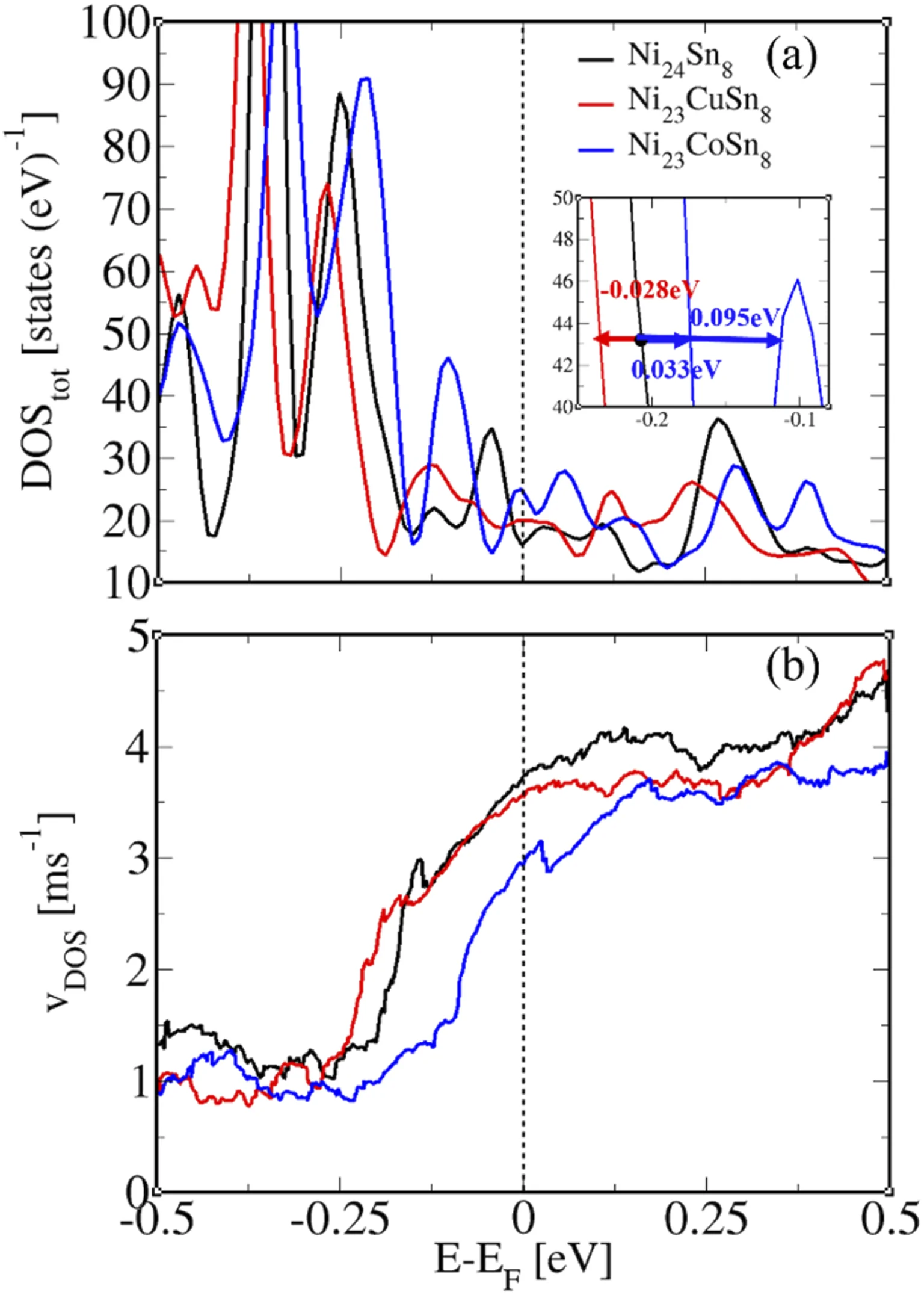
(a) Calculated total GGA-PBE density of states, where the zoom-in inset shows the Cu substituted compound's DOS shifting further away from *E*_F_ by an additional −0.028 eV, and the Co substituted compound's DOS shifting closer to *E*_F_ by 0.033 eV in energy (*E*_F_ = 0 eV). (b) Band structure velocities for the three materials under consideration, calculated using ElecTra.^33^ The velocities undergo a large drop, reflecting the less dispersive flattened out hole-like bands below *E*_F_.

The total DOS-weighted average of the band velocities calculated using ElecTra^33^ is shown in Fig. 4b, and is computed as:3
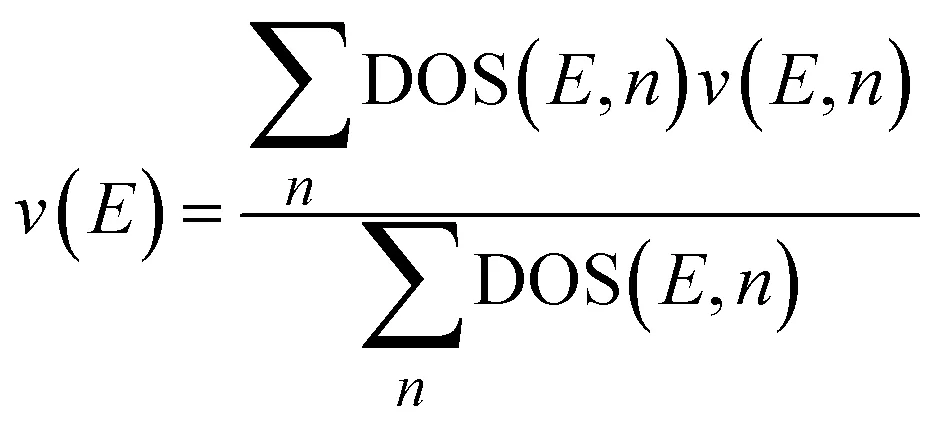
For energies above *E*_F_, the band velocities have high values, typical for dispersive bands. The velocities drop significantly for energies below *E*_F_ for all three materials, indicating a much lower *v*(*E*) associated with the flat bands. All three materials have comparable *v*(*E*). The hole-like bands include flattened out bands and also dispersive bands, whereas the DOS-weighted *v*(*E*) weights the large DOS flat bands more, leading to the observed net reduction in velocities below *E*_F_. For chemical design this means that dispersive bands below *E*_F_ are acceptable and are usually present in metals, but heavy bands will increase scattering, which creates the sizeable variation in transport to observe *S* enhancements.

Aware of the limitations of GGA-PBE we have additionally investigated the effect of Coulomb interactions within the GGA+U framework by employing a Hubbard parameter of *U* = 1 eV as a first-order assessment of the flat-band features in these systems (more advanced treatments such as HSE06 are computationally prohibitive for the large unit cells of 32 atoms). It turns out that the flat bands shift slightly further below *E*_F_ compared to the corresponding GGA band structures flat bands' positions, however, the specific *U* used also results in uncertainty. A comparison of the GGA and GGA+U band structures and DOS for all three systems is provided in Fig. S22 and S23 in the SI together with appropriate discussions. In the case of PBE, the shift between the d-orbitals’ raising edge from the pristine case is −0.028 eV and +0.033 eV for Cu and Co, respectively. In the case of GGA+U these change to −0.046 eV and +0.051 eV for Cu and Co, respectively, which is larger than in the PBE approach. However, due to the larger uncertainties in both methods and experiment, these values are only used as guidance in the two-band model calculations.

Furthermore, the influence of spin–orbit coupling (SOC) and spin polarization is examined to determine whether the flat-band features and band geometries are affected by relativistic or magnetic effects. To reduce the computational cost, these tests were performed on the primitive cells of the pristine systems, rather than on the larger supercells. The results (see Fig. S24 in SI) show that the flat-band features remain essentially unchanged, with only minor band splitting observed at a few *k*-points with SOC. In addition, the spin-polarized DOS reveals no evidence of magnetic ordering in any of the systems. A detailed discussion of these results is presented in Section II.2 in the SI.

### 2.4 Parabolic two-band model development for Ni_3_Sn

To model these materials at first-order level, a basic parabolic two-band model was employed as shown in Fig. 5a. This model includes a light electron-like and a heavy hole-like band, with an energy overlap. The model description is based on the PBE computed DFT band structures. The DFT band structures are composed of multiple overlapping bands, thus it is not straightforward to use a simple model that can provide first order understanding.

**Fig. 5:**
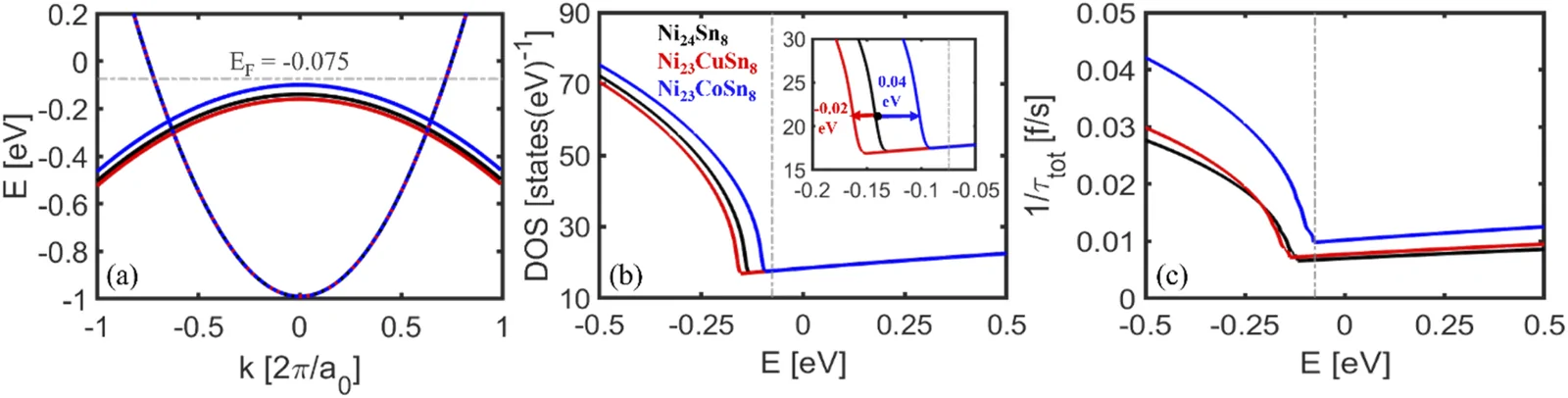
(a) Overlapping parabolic two-band model used to model the transport properties. (b) Total DOS from the parabolic bands for the three materials under consideration. (c) Total scattering rates for the two-band model assuming intra- and inter-band scattering for both ADP and ODP mechanisms.

However, the DFT DOS and the band velocities can drive the way into forming an informative two-band model as follows: (1) the choice of the heavy and light band effective masses is such that their band velocities are mapped to those of the DFT bands as shown in Fig. S16d–f. The overlap of the two bands is set to map the parabolic band velocities as much as possible to the DFT velocities in the electron-like region. This is an effective way for the two-band model to map the curvatures of the DFT bands. (2) Then for each band a degree of degeneracy is used in order to map the magnitude of DOS in each hole-like/electron-like region as shown in Fig. S16a–c. The relative energy positions of the hole-like bands as well as their energy shifts upon doping with Cu and Co are kept similar to the trends observed in the DFT-DOS and DFT velocity trends, but adjusted slightly in order to match the *S*(*T*) measurements as close as possible. Combined, the total fitting DOS for the three materials is shown in Fig. 5b and resembles the DFT DOS in Fig. 4a. The values for the flat band shift from the pristine material used to match the experimental *S*(*T*) are −0.02 eV and +0.04 eV for Cu/Co (the latter somewhat in between PBE and GGA+U). The overlap of the two bands is an important design fitting parameter. The larger it is, the higher the electron-like carrier velocities are in the energy region right after the flattened band region ends, which allows for the high metallic conductivity.

In DFT the hole-like bands include both flattened and more dispersive bands, but the main effect is the strong inter-band scattering of the electron-like dispersive bands, for which inclusion of only a flattened hole-like band is sufficient. The final ingredient in the model, therefore, is the scattering of carriers below *E*_F_ into the flatter band. To evaluate the scattering rates, a simplified first order approximation *i.e.* elastic, acoustic deformation potential (ADP) for small range transitions, and inelastic optical deformation potential (ODP) for long range transitions including intra- and inter-band scattering was used. The corresponding total scattering rates are shown in Fig. 5c. The full details on scattering and transport modelling are provided below in Section 4.3, and the relevant parameters are listed in Table S7 in the SI. Note that *ab initio* calculations for scattering times for the alloys under investigation is computationally prohibitive because they would require the calculation of matrix elements, which is essentially impossible for the large 24 atoms alloy unit cells considered. The main effect, however, comes from scattering being proportional to DOS, which is something originating from the established Fermi's Golden rule and applicable widely. Note that under ADP/ODP scattering, the scattering times are proportional to the inverse DOS, which cancels the DOS in *G*(*E*). Thus, *G*(*E*) essentially follows the velocity energy trends as shown in Fig. S17 and S18 for the three materials considered, which can be used as a simple first order indication of sharp changes in electronic conductivity and increase in *S*. Indeed, the characteristic output of this two-band model and the ADP/ODP intra- and inter-band scattering is the sharp increase in *G*(*E*) around *E*_F_. It is well known that sharp changes in *G*(*E*) result in large increases in *S*.^17^ Plotted as a function of temperature, alongside with the experimental data, the TE coefficients, *S*, *σ*, and PF obtained this way, provide a very good qualitative and quantitative agreement with experimental data, in particular considering the simplicity of the model, as shown in Fig. 2.

## 3. Discussion

The main outcome of this study is that like the bandgap in semiconductors, utilising a mixture of heavy and light bands and selective carrier scattering, is a robust route to achieving large *S* and PF. This is demonstrated for Ni_3_Sn, where such mixture exists, and Co substitution optimises the alignment of *E*_F_ near that heavy and light band intersection. This supports PFs up to 5 mW m^−1^ K^−2^ with modelling suggesting values up to 8–9 mW m^−1^ K^−2^ should be possible in samples with optimised *S* ∼65 µV K^−1^ through control of the carrier density (Fig. S21). These values are large compared to typical semiconductor thermoelectrics. The *zT* of these metals, however, remains low for the current materials. The only strategy to significantly improve performance is through increasing *S*. This can be seen from *zT* = *S*^2^/*L* for ideal metals. Using the observed *S*_max_ = 55 µV K^−1^ and *L* = 1.9 × 10^−8^ V^2^ K^−2^ yields *zT* = 0.16, which is around twice the experimental value (Fig. 2f). This reflects the near equal electronic and lattice thermal conductivity contributions. However, unlike semiconductor thermoelectrics, there is no strong benefit to lowering *κ*_lat_. Instead, the only way to substantially improve *zT* in metallic thermoelectrics is to increase *S.* For example, *S* = −140 µV K^−1^ corresponds to *zT* = 1 using *L* = 1.9 × 10^−8^ V^2^ K^−2^. From the two-band model, higher *S* is driven by increasing the mismatch in band dispersion and degeneracy between electron- and hole-like bands to create step-changes in DOS that underpin increased selective scattering. This ultimately creates an asymmetry in the transport distribution function *G*(*E*), with large *S* if *E*_F_ is near the step-change. Chemically, this requires a combination of weaker (localised d-bands) and stronger bonding, involving main group elements, yielding more dispersive bands. In the very high PF Ni–Au alloys (*S*_max_ = 80–90 µV K^−1^) this bonding (band) mismatch is stronger than in Ni–Cu, which is comparable to the Ni_3_Sn system in terms of *S* and PF values. From the DFT calculations, the quantity that most directly predicts *S* is the step-change in the DOS-weighted velocity distribution as shown in Fig. 4. Here, the Co doped material has the step-change closest to *E*_F_ and consequently this material has the highest observed *S*. Note that the sole dependence of *zT* on *S* might make it appear as that the conductivity *σ* is not essential and there is no need to optimize it. This is deceiving: the much larger conductivity of the dispersive bands to the right of *E*_F_, compared to the low conductivity in the flat band region, which induces excess scattering, is what creates and enhances the anisotropy in *G*(*E*) that increases *S*. In that sense, the velocity quantity discussed above, which directly links to conductivity, may provide a good way to screen DFT materials databases for promising candidate materials.

Our work complements the earlier study on In substituted Ni_3_Sn_1−*x*_In_*x*_ which also removes carriers and is comparable to Co doping.^19^ That study had a similar *S*_max_, supporting our conclusion that the position of *E*_F_ is the main factor that leads to performance enhancement. Texturing and anisotropic microstrain are characteristics of the initial series of materials. Nevertheless, these samples show systematic changes in properties, demonstrating that the impact of microstructure is not as significant as the changes in chemical composition (*E*_F_ tuning). The properties are also in good agreement with the large Ni_2.9_Co_0.1_Sn ingot, which is not textured, confirming the dominant influence of chemical composition.

Beyond thermoelectric performance, the thermal transport in these materials is also of interest. Specifically, separating *κ*_lat_ and *κ*_el_ is difficult in metals and requires an accurate value for *L*, *i.e.* using the free electron value readily leads to severely underestimated or even negative *κ*_lat_. A realistic estimate of *L*(*T*) is readily extracted from the two-band parabolic model (see SI for details) affording a better estimate of *κ*_lat_ as shown in Fig. 2. Additionally, the electronic contribution to the heat capacity is significant (∼20% at 800 K) and it is not appropriate to use the Dulong Petit value, which leads to an underestimate of *κ.* Finally, the rising and then decreasing *κ*(*T*) of the Ni_3_Sn parent material is of interest. The same *κ*(*T*) trend was found in the Ni_3_Sn_1−*x*_In_*x*_ study, with In substitution leading to a linear increasing *κ*(*T*) as also observed for the Co and Cu doped samples. It therefore appears that chemical doping has a strong influence on both magnitude and temperature dependence of *κ*(*T*).

Theoretical calculations using a two-band parabolic model can at first order capture the trends in experimental data with good quantitative agreement. The model simply uses two overlapping parabolic bands, with the overlap energy region of the bands, the masses of the bands (band curvature) and their degeneracy, *E*_F_ and the intra-/inter-band deformation potentials being the model parameters. The band masses are set by fitting to the DFT band velocities, while their degeneracy is set to map the DFT DOS in the flat and dispersive regions. The deformation potentials are then further slightly adjusted from a basis value to obtain a decent map to experiment for each material. In that respect, the model is not phenomenological, but is based on *ab initio* calculations. The main conclusion from the modelling is that the dominant effect is the presence of flattened-out d-orbital hole-like bands, which yield a high density of states below *E*_F_. This significantly increases scattering of the hole-like carriers below *E*_F_, resulting in *S* values that are large for metals. The main effect that leads to the measured observations of reduced *S* in the Cu substituted material and the increased *S* in the Co substituted material (in absolute terms), is the shift in the sharp DOS region with respect to the *E*_F_. The Cu substitution moves the sharp edge farther away and the Co substitution moves it closer to *E*_F_. In fact, Fig. S19 shows that using a common set of input parameters for all three compounds, other than the position of the sharp DOS with respect to *E*_F_, captures the main effects. Thus, the main parameter in describing the experimental results in Fig. 2 is the position of *E*_F_, with the other parameters being of secondary importance for the enhanced *S*. Hence, *S* enhancement in a system with flat d-bands and dispersive bands near *E*_F_ is controlled by tuning the carrier density, which is done here by using Co/Cu substitution in Ni_3_Sn. The dominance of carrier density explains why the observed properties in this study are similar to Ni_3_Sn_1−*x*_In_*x*_, with any differences to the bands caused by the chemical substitutions apparently less important, in agreement with the conclusions from the model. Model deviations from experiment, especially in the Co substituted materials, can be attributed to band complexities and scattering complexities, for which our model assumes two-band isotropic, elastic acoustic phonon scattering simplifications. For example, if a third dispersive hole like-band is added in the model, better match to the *S*(*T*) can be reached (not shown). Further scattering considerations regarding the effect of inelastic phonon scattering processes could possibly have additional effect, as well as Zener tunnelling around band crossings (although such Dirac-like bands in our case are further from *E*_F_).^19^ The main point, however, is that the simplicity of the two-band model captures the *S* enhancement and is preferred for this analysis. Also note that, as shown in Fig. 5b, the shifts in the high DOS region below *E*_F_ used to fit the experimental data, are comparable for all three systems, but do not coincide precisely with the corresponding DOS energy positions predicted by DFT. This illustrates the differing predictive capabilities and inherent limitations of both DFT calculations and simple parabolic models, when compared with experimental results. Nevertheless, the relative shift of the high DOS region explains the *S* trends at first order. Additionally, the transport coefficients of these materials as a function of chemical potential near room temperature are shown in Fig. S21. They indicate large PFs for the Co substituted material at ∼−0.075 eV, which has the DOS and the *G*(*E*) sharp region moving closer to *E*_F_. This suggests that further improvements in properties would be possible by controlling the Co content experimentally.

To conclude, carrier filtering by scattering is a robust effect that occurs in metallic systems with an appropriate band structure that has the Fermi level near to a peak in the electronic density of states. The model approach of two overlapping parabolic bands provides a timely and significant contribution to understanding band engineering of metallic systems, which opens a new field for materials design. In Ni_3_Sn this leads to power factors that are comparable to good semiconductor thermoelectric materials. Further exploration of intermetallic compound thermoelectrics, including further manipulation of Ni_3_Sn, is a highly promising avenue towards broadening the range of materials available, with potential implications for sustainability and resource use.

## 4. Methods

### 4.1 Materials synthesis and characterisation

Ni_3−*x*_M_*x*_Sn (M = Cu, Co) samples were prepared on a 5 g scale by arc melting. Elemental slugs purchased from Alfa Aesar (Ni, Co, Cu) with purity of ≥99.995% and shots purchased from thermoscientific (Sn) with a purity of ≥99.999%, were weighed, and arc melted several times using an Edmund Bühler MAM-1 to ensure homogeneity. The resulting ingot was shaker milled (20 Hz, 2 h, MM400 Retsch). About 2.5 g of powder was hot pressed (1000 °C, 10 bar, 30 min) to obtain 13 mm diameter discs of ∼2 mm thickness. A large sample of Ni_2.9_Co_0.1_Sn was prepared to measure all thermoelectric properties parallel (‖) and perpendicular (⊥) to the hot-pressing direction. 20 g of material was melted using a tetra arc instrument from Techno Search (Japan). About 14 g was hot pressed three times at 1000 °C to obtain an ∼8 mm tall ingot with near theoretical density.

All samples were initially characterised using powder X-ray diffraction (XRD). Data was collected over 60 minutes in a range of 10–100° using diffractometer PANalytical Empyrean from Malvern PANalytical with an X'Calibration linear detector. The measurements were performed with Cu-K_α1_ monochromated radiation in reflection mode. For analysis Rietveld refinements were performed using general structure analysis system-II (GSAS-II).^34,35^ For scanning electron microscopy (SEM) and energy dispersive X-ray spectroscopy (EDS) all samples were polished with SiC sandpaper, then with subsequent 15 µm, 5 µm and 1 µm diamond polish pastes. Measurement and data handling was done using a tungsten filament JEOL JSM-IT200 scanning electron microscope with JEOL DrySD EDS detector, containing both secondary electrons and back scattered electrons detectors.

The electrical resistivity *ρ* and absolute Seebeck coefficient *S* were measured using a Linseis LSR-3 instrument on bar-shaped specimens (∼1.5 × 2 × 7–10 mm^3^), under a static He atmosphere. Longer equilibration times were used to ensure a stable temperature gradient during the *S* measurement. Two cycles were measured to ensure reproducibility. The thermal diffusivity *α* was measured using a Linseis LFA-1000 laser flash instrument under dynamic vacuum conditions. For the initial series of samples measurements were done on the hot-pressed discs of 13 mm diameter and ∼2 mm thickness. The larger Ni_2.9_Co_0.1_Sn ingot was measured in its entirety for the parallel to pressing direction. For the perpendicular direction a smaller 3 × 3 mm^2^ square of 1 mm thickness was used. The thermal conductivity *κ* was then calculated from the equation *κ* = *C*_p_*αρ*_d_, with measured heat capacity *C*_p_ and measured density from physical dimensions *ρ*_d_. Porosity correction was performed for all samples below theoretical (crystallographic) density *ρ*_cry_ using *κ*_corrected_ = *κ*/(1 − (4/3)*ϕ*), where *ϕ* = (1 − *ρ*_d_/*ρ*_cry_).^36^ Measured densities are listed in Table S4. The *C*_P_ was measured using Differential Scanning Calorimetry (DSC) on a Netzsch DSC 04 F1 Phoenix instrument with dynamic nitrogen flow.

The longitudinal *ν*_l_ and transverse *ν*_t_ velocities of sound were measured on hot-pressed discs using the ultrasound method using an Evident Scientific Epoch650 instrument. The transducers were coupled using SWC-2 couplant solution (Evident Scientific). Elastic moduli were calculated using established protocols.^32,37^

### 4.2 DFT calculation details

The electronic structures of Ni_24_Sn_8_, Ni_23_CuSn_8_, and Ni_23_CoSn_8_ were computed using density functional theory (DFT) as implemented in Quantum Espresso.^38^ These bandstructures were obtained from a 2 × 2 × 1 supercell (32 atoms) of pristine Ni_3_Sn, where 4.2% substitution with Cu and Co, respectively, was introduced by replacing one Ni out of the 24 Ni atoms. The PWscf package was used to solve the self-consistent Kohn–Sham equations using plane-wave (PW) basis sets.^39^ The kinetic-energy cutoff for the plane-wave basis was set to 60 Ry, while the charge-density cutoff was taken as eight times higher to ensure numerical accuracy. The atomic cores were described using RRKJ-type ultrasoft pseudopotentials (USPP). The electronic exchange–correlation effects were treated within the generalized gradient approximation (GGA) using the Perdew–Burke–Ernzerhof (PBE) functional,^40–43^ as used by many other metallic alloys studies.^44^ For the self-consistent field (SCF) calculations, a uniform 20 × 20 × 20 *k*-point grid was used, whereas a denser 40 × 40 × 40 *k*-grid was employed for non-self-consistent (NSCF) calculations to obtain accurate density of states results. All the supercell structures were first relaxed before performing the electronic structure and DOS calculations. A force convergence threshold of 0.001 a.u. and pressure convergence threshold of 0.5 kbar was used. The Marzari–Vanderbilt smearing scheme was applied to treat fractional orbital occupancies appropriate for metallic systems.^45^ Moreover, to check the geometry and position of the flat bands in these systems further accuracy tests were applied *i.e.* DFT+U (with *U* = 1 eV as a first order indication), SOC, and spin-polarization effects for all three systems.

### 4.3 Parabolic two-band transport model details

In order to emphasize the change in the d-orbital peak with respect to *E*_F_ as the main reason for the observed *S*(*T*), all other band parameters are kept the same for all three materials, *i.e.* band effective masses (thus the slight mismatch in velocities to DFT velocities in Fig. S16 in SI), band degeneracies, positioning of the light electron-like band, and also kept a common *E*_F_ at −0.075 eV. Note that this position was chosen to best match the transport data and is not from DFT. The only band-related parameter varied is the band edge of the heavy hole-like band for the three material cases.

After obtaining the parabolic DOS, the scattering rates are calculated as a next step in the model using deformation potential theory, including both acoustic phonon scattering (ADP) and optical phonon scattering (ODP). Although the precise details of these are unknown for the specific materials, the intent is to capture all possible scattering features that can be present, *i.e.* both elastic and inelastic processes, and processes that involve states that are nearby and far away in the Brillouin zone. ADP captures elastic processes and ODP captures inelastic processes involving transitions from states nearby and far from each other in the Brillouin zone, respectively.

The energy dependent scattering rates for ADP are:4
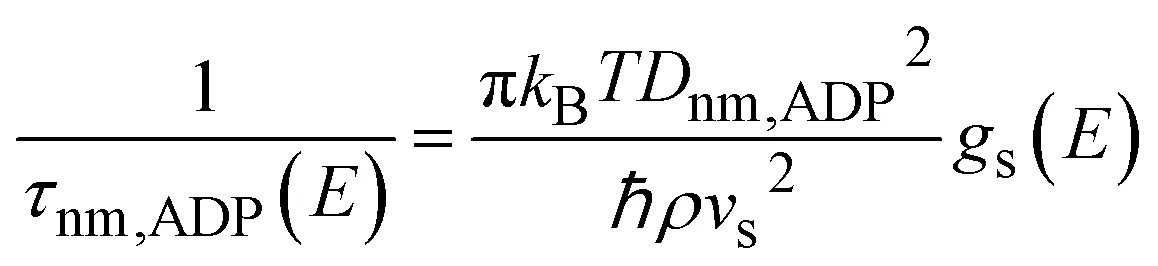
Here, *D*_nm_ is the deformation potential for both the intra-band, *n = m* (*D*_intra_) and inter-band *n* ≠ *m* (*D*_inter_) transitions, although here the same *D* value is used in a specific material for both transitions for convenience in reduced number of model parameters. *v*_s_ is the sound velocity, *ρ* is the mass density, and their values used in the model are the crystallographic values for each system, as shown in Table S4 in the SI, while *g*_s_ is the final scattering density of states, where *s* = *n*, for intra-band and *s* = *m* for inter-band transitions.

The overall ADP scattering rate from an initial state at energy *E* and band *n* is then computed by adding the intra- and inter-band transition rates as:5
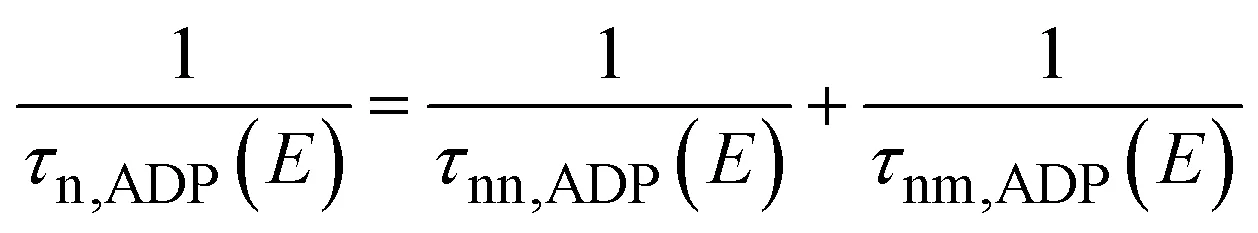


Similarly, to include the ODP scattering rates, which can allow for small and large *k*-space transitions, the typical rate equations are used as:6
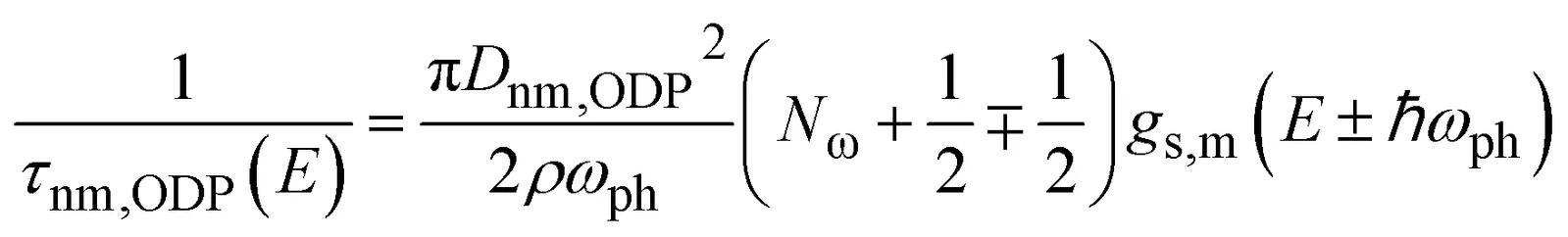
Here, *D*_nm,ODP_ is the optical deformation potential (use of the same value for both intra- and inter-band processes for a specific material), *ω*_ph_ is the optical phonon frequency, *N*_ω_ is the Bose–Einstein distribution function, and *g*_s_(*E* ± *ħω*_ph_) is the final scattering density of states for the emission/absorption processes. The treatment of intra- and inter-band transitions is as above for ADP.

Finally, the total scattering rate (ADP + ODP) is computed using Matthiessen's rule as:7
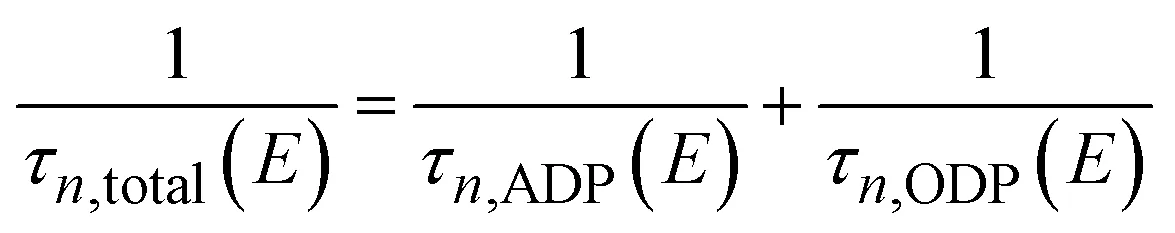


Note that in order to reduce the number of fitting parameters and focus more on band shift changes, the same phonon frequency is used for all materials and the same *D*_ADP_ and *D*_ODP_ values for both intra- and inter-band transitions in a certain material. The magnitude of the ADP and ODP scattering rates are kept similar in all material cases. These values are slightly varied across materials by less than 10% to achieve better match to the experimental conductivity. The corresponding total scattering rates are shown above in Fig. 5c.

The model fitting parameters are shown in Table S7 in the SI. Importantly, they are not arbitrary, but their band related values, which determine the *S* – *e.g.*, a heavier hole band leading to a larger density of states and stronger scattering, are based on the DFT calculations, and their scattering related parameters, which determine the conductivity, are within physically reasonable range compared to common materials.

The scattering rates are then used with the transport distribution function (TDF) to determine the transport coefficients, which is given here as *G*(*E*):

8 *G*(*E*) = *τ*_*n*_(*E*)*v*^2^(*E*)*g*(*E*)

where *τ*_*n*_(*E*) is the total relaxation time (inverse of scattering rate). The transport coefficients (*σ* and *S*) are then evaluated by integrating over energy using this TDF term *via* the linearized Boltzmann transport equation (BTE) as^46^9
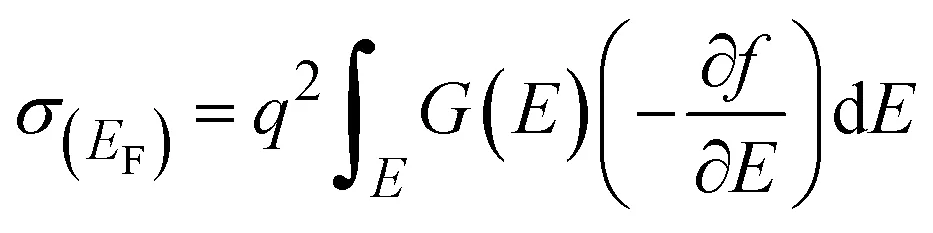
10
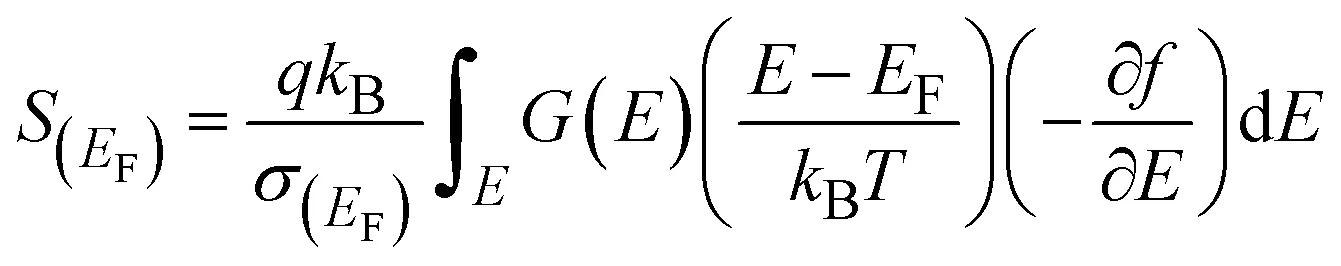

11 PF = *σ*_(*E*_F_)_*S*_(*E*_F_)_^2^

where *q* is the electronic charge, *f* is the fermi distribution function, *k*_B_ is the Planck's constant, *E*_F_ is the Fermi energy level.

## Conflicts of interest

There are no conflicts of interest to declare.

## Supplementary Material

TA-014-D6TA01928E-s001

## Data Availability

Data underpinning this research is available *via* the University of St Andrews data Repository, https://doi.org/10.17630/ec23eb15-6d34-4fb7-9d59-818f327e106b. Supplementary information (SI): supporting X-ray diffraction, tables with crystallographic information, velocity of sound data, scanning electron microscopy and elemental mapping, thermoelectric cycling data, heat capacity, additional DFT and band modelling data. See DOI: https://doi.org/10.1039/d6ta01928e.
