# Supplementary material for: Enhanced power factor by transition metal substitution in the thermoelectric Kagome metal Ni3Sn
Source: J Mater Chem A Mater. 2026 Sep 25;14(65):45040–51. doi: 10.1039/d6ta01928e (PMC13655886; doi:10.1039/d6ta01928e)
Supplement: Supplementary file 2 [file TA-014-D6TA01928E-s001.pdf]

Supplementary Information (SI) for Journal of Materials Chemistry A.
This journal is © The Royal Society of Chemistry

Supporting Information for:

Enhanced power factor by transition metal substitution in the thermoelectric Kagome metal Ni_3Sn

Kim-Isabelle Mehnert-Birk,[1] Saff E. Awal Akhtar,[2] Blair F. Kennedy,[1] Ankit Kumar[2], Neophytos Neophytou[2#] and Jan-Willem G. Bos[1*]

[1] *EaStCHEM School of Chemistry, University of St Andrews, North Haugh, St Andrews, KY16 9ST, United Kingdom*

[2] *School of Engineering, University of Warwick, Coventry, CV4 7AL, United Kingdom*

[#] N.Neophytou@warwick.ac.uk
[*] j.w.g.bos@st-andrews.ac.uk

I – Supporting Experimental Data

Table S1: Lattice parameters and cell volume for $Ni_{3-x}M_xSn$ samples from XRD data collected on powdered hot-pressed discs.

composition	*a* (Å)	*c* (Å)	*V* (Å^3)
Ni_3Sn[1]	5.296	4.247	
Ni_3Sn	5.2959(2)	4.2469(1)	103.15(1)
$Ni_{2.9}Cu_{0.1}Sn$	5.2976(2)	4.2544(1)	103.40(1)
$Ni_{2.9}Co_{0.1}Sn$	5.2995(2)	4.2485(1)	103.33(1)
$Co_{0.1}$ □ & □	5.2959(3)	4.2471(1)	103.16(1)
$Ni_{2.8}Co_{0.2}Sn$	5.2983(2)	4.2460(1)	103.23(1)

Table S2: March-Dollase (MD) (0 0 2) texture parameters for $Ni_{3-x}M_xSn$ samples from XRD data collected on surface of hot-pressed and powdered hot-pressed disks. Microstrain parameters are from data on powdered disks.

composition	MD-disc surface	MD powder	Equatorial Strain (%)	Axial strain (%)
Ni_3Sn	0.59	0.72	0.68	0.36
$Ni_{2.9}Cu_{0.1}Sn$	0.72	0.96	0.57	0.44
$Ni_{2.9}Co_{0.1}Sn$	0.73	0.87	0.66	0.41
$Co_{0.1}$ □	0.87	0.83	0.46	0.38
$Co_{0.1}$ Ʇ	0.99	0.83	0.46	0.38
$Ni_{2.8}Co_{0.2}Sn$	0.74	0.66	0.75	0.39

Table S3: Nominal compositions of $Ni_{3-x}M_xSn$ and composition derived from EDS elemental maps.

nominal composition	EDS composition
Ni_3Sn	$Ni_{2.78(1)}Sn_{1.00(1)}$
$Ni_{2.9}Cu_{0.1}Sn$	$Ni_{2.66(1)}Cu_{0.08(1)}Sn_{1.00(1)}$
$Ni_{2.9}Co_{0.1}Sn$	$Ni_{2.66(1)}Co_{0.09(1)}Sn_{1.00(1)}$
$Ni_{2.8}Co_{0.2}Sn$	$Ni_{2.49(1)}Co_{0.17(1)}Sn_{1.00(1)}$

Table S4: Measured, crystallographic and % density of the $Ni_{3-x}M_xSn$ hot-pressed discs characterised in this manuscript.

composition	measured □ (g cm^{-3})	crystallographic □ (g cm^{-3})	% density
Ni_3Sn	9.17	9.45	97
$Ni_{2.9}Cu_{0.1}Sn$	8.32	9.48	88
$Ni_{2.9}Co_{0.1}Sn$	8.89	9.48	94
Large ingot	9.47	9.48	100
$Ni_{2.8}Co_{0.2}Sn$	8.57	9.49	90

Table S5: Measured longitudinal $\square_l$ and transverse $\square_t$ velocity of sounds for $Ni_{3-x}M_xSn$ samples, and derived parameters, including mean velocity of sound $\square_m$, Poisson ratio $\square_p$, Shear Modulus *G*, Bulk Modulus *B*, Pugh's ratio *B*/ *G*, Young modulus *Y*, Grüneisen parameter γ and Debye temperature $\square_D$.

composition	Ni_3Sn	$Ni_{2.9}Co_{0.1}Sn$	$Ni_{2.8}Co_{0.2}Sn$	$Ni_{2.9}Cu_{0.1}Sn$
$\square_t$ [m s^{-1}]	2706(7)	2764(16)	2663(2)	2489(26)
$\square_l$ [m s^{-1}]	5357(13)	5552(11)	5199(14)	5383(15)
$\square_m$ [m s^{-1}]	3033(7)	3101(16)	2983(2)	2804(28)
□[g cm^{-3}]	9.455	9.478	9.489	9.484
$\overline{M}$ [u atom^{-1}]	73.70	73.70	73.71	73.82
V_0 [Å^3 atom^{-1}]	12.94	12.91	12.90	12.93
$\square_p$	0.329(2)	0.335(3)	0.322(1)	0.364(4)
B [GPa]	179.0(6)	196(1)	166.7(3)	196(2)
G [GPa]	69.2(3)	72.4(8)	67.3(1)	58(1)
B/ *G*	2.59(2)	2.70(3)	2.48(6)	3.34(8)
Y [GPa]	184(1)	193(2)	177.9(3)	160(3)
γ	1.96(1)	2.01(2)	1.92(2)	2.25(3)
$\square_D$ (K)	384.6(9)	393(2)	378.7(3)	355(4)
$\square_{lat}$ [W m^{-1} K^{-1}]	10.5(2)	10.7(3)	10.5(1)	6.3(3)

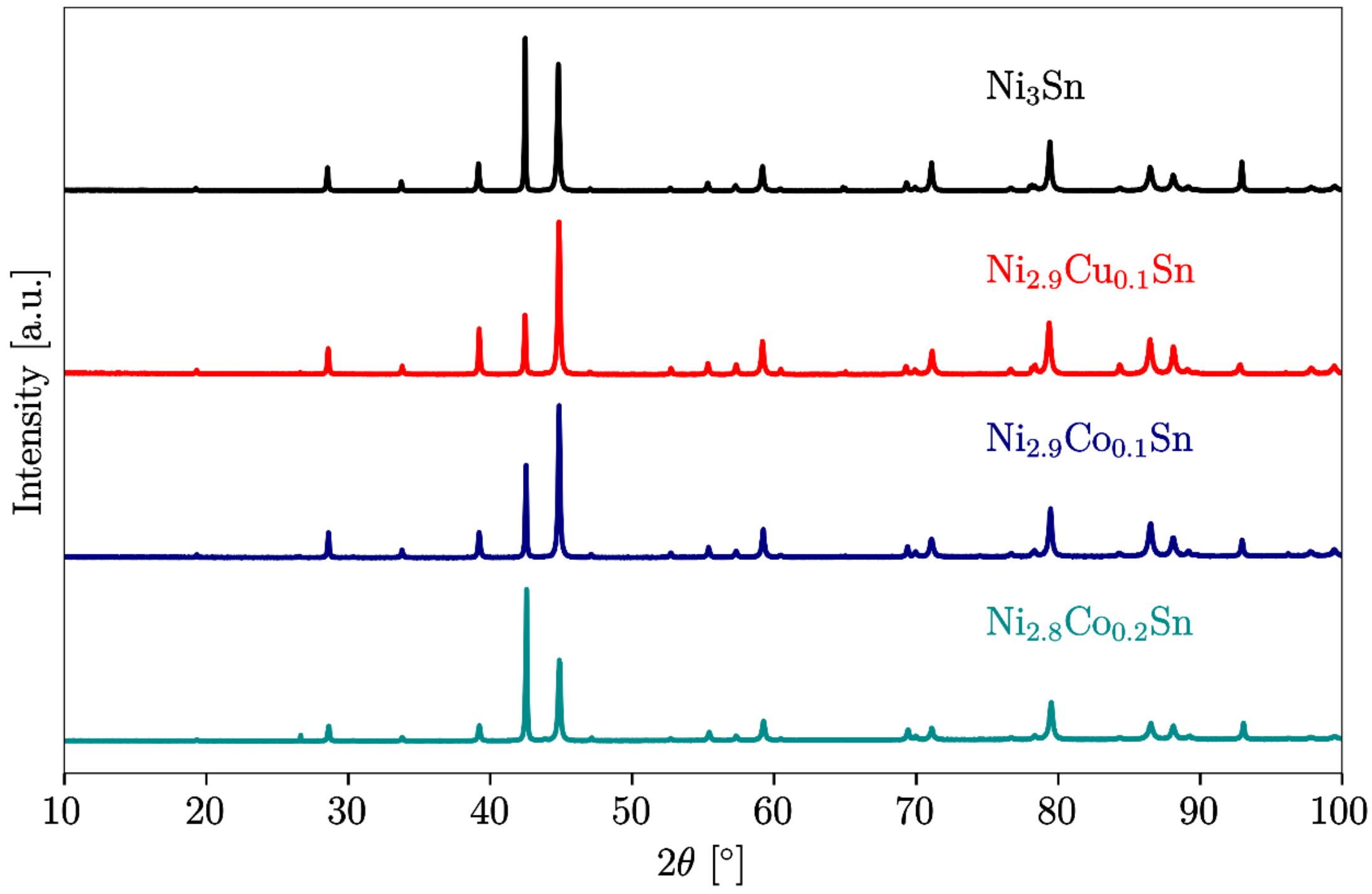

Figure S1: XRD data for $Ni_{3-x}M_xSn$ samples collected on powdered hot-pressed discs.

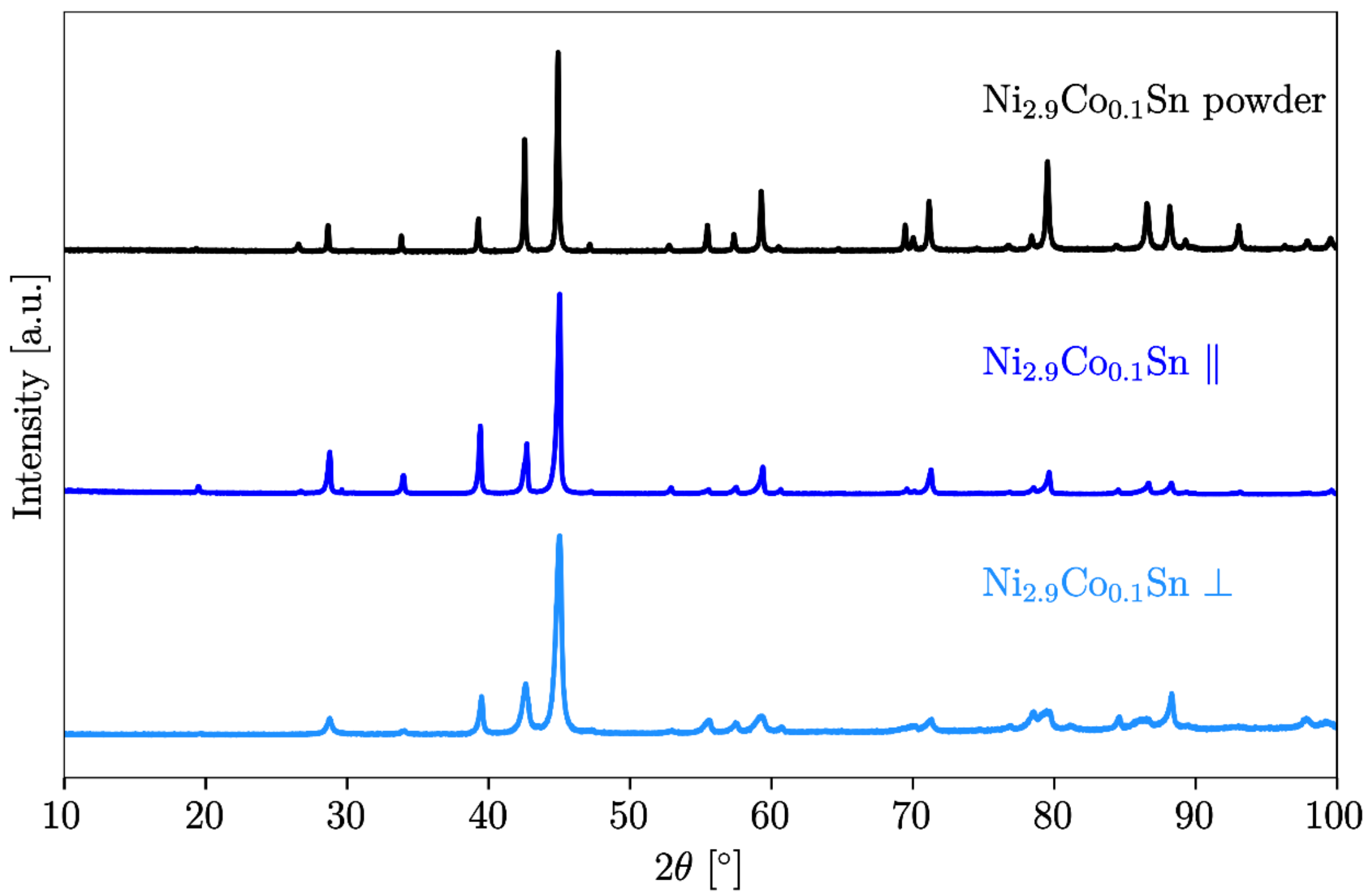

Figure S2: XRD data for $Ni_{2.9}Co_{0.1}Sn$ sample collected on hot pressed (hp) disc parallel □ and perpendicular Ʇ to pressing direction and on a powder sample from the disc.

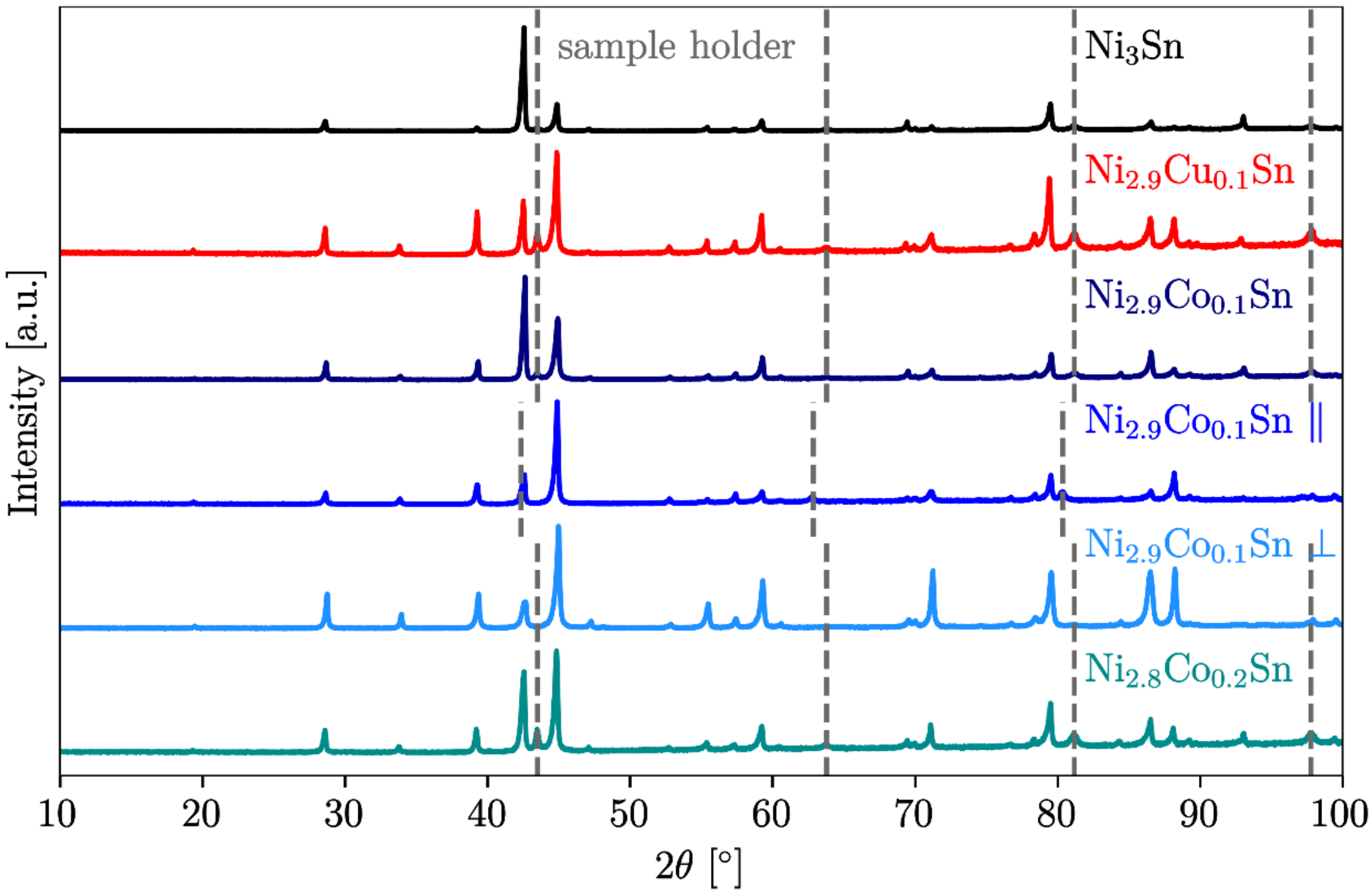

Figure S3: XRD data for $Ni_{3-x}M_xSn$ collected on LSR bars after measurement and after 24 months storage for Ni_3Sn, $Ni_{2.9}Cu_{0.1}Sn$, $Ni_{2.9}Co_{0.1}Sn$ and $Ni_{2.8}Co_{0.2}Sn$ and 7 months for $Ni_{2.9}Co_{0.1}Sn$ || and $Ni_{2.9}Co_{0.1}Sn$ Ʇ. Samples were stored in a closed sample vial in air.

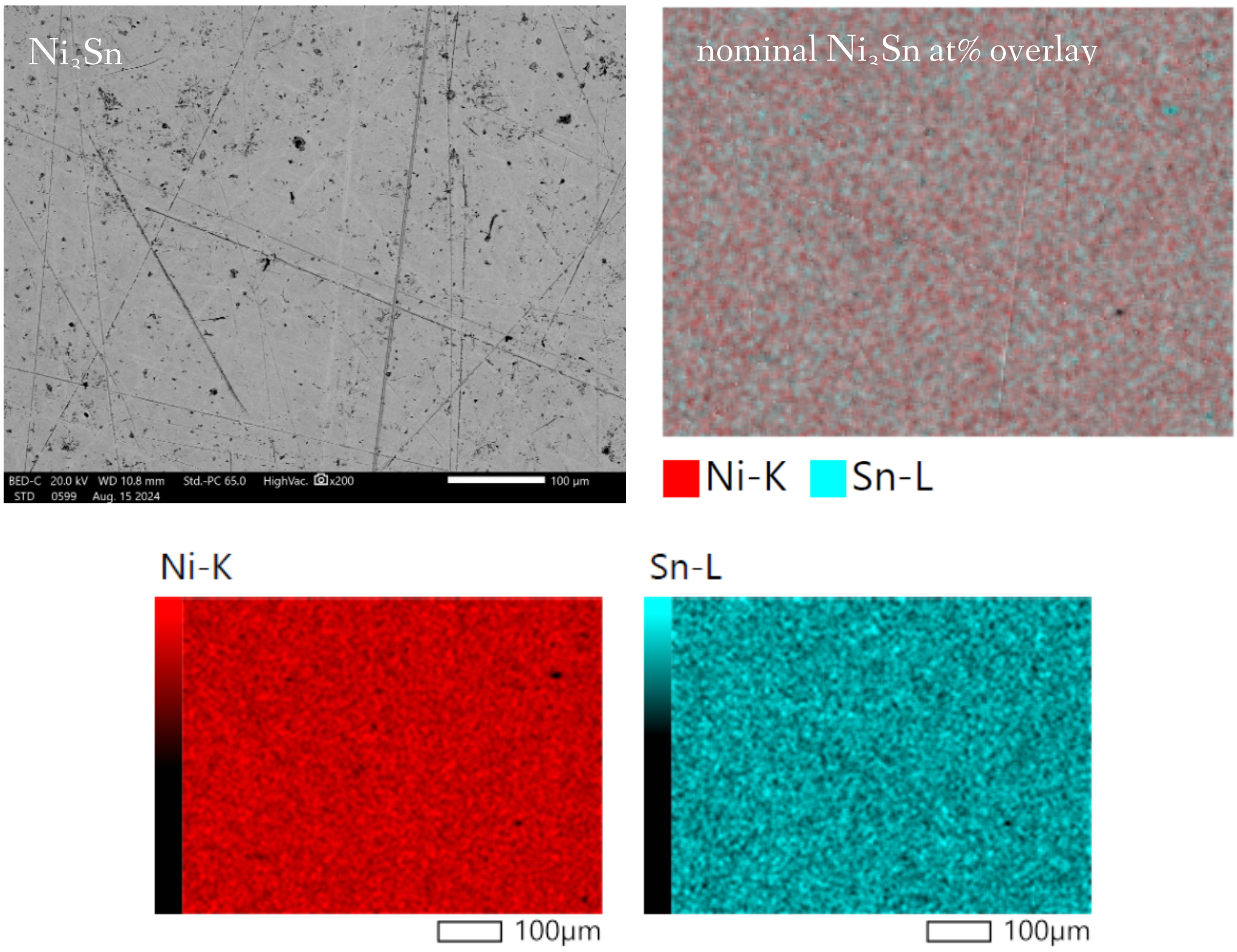

Figure S4: Back scattered detector SEM image, at% composite and individual elemental at% maps for Ni_3Sn.

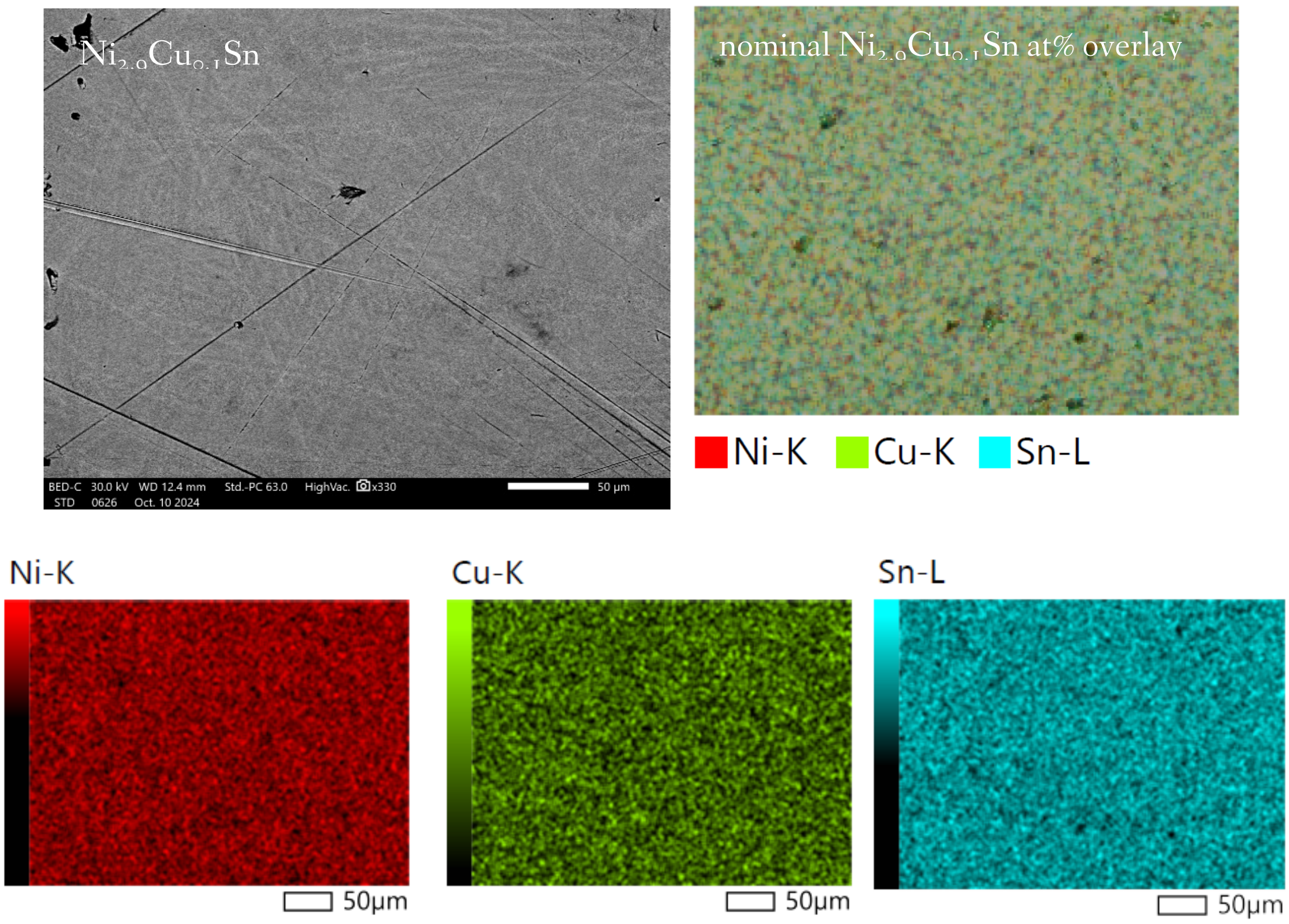

Figure S5: Back scattered detector SEM image, at% composite and individual elemental at% maps for $Ni_{2.9}Cu_{0.1}Sn$.

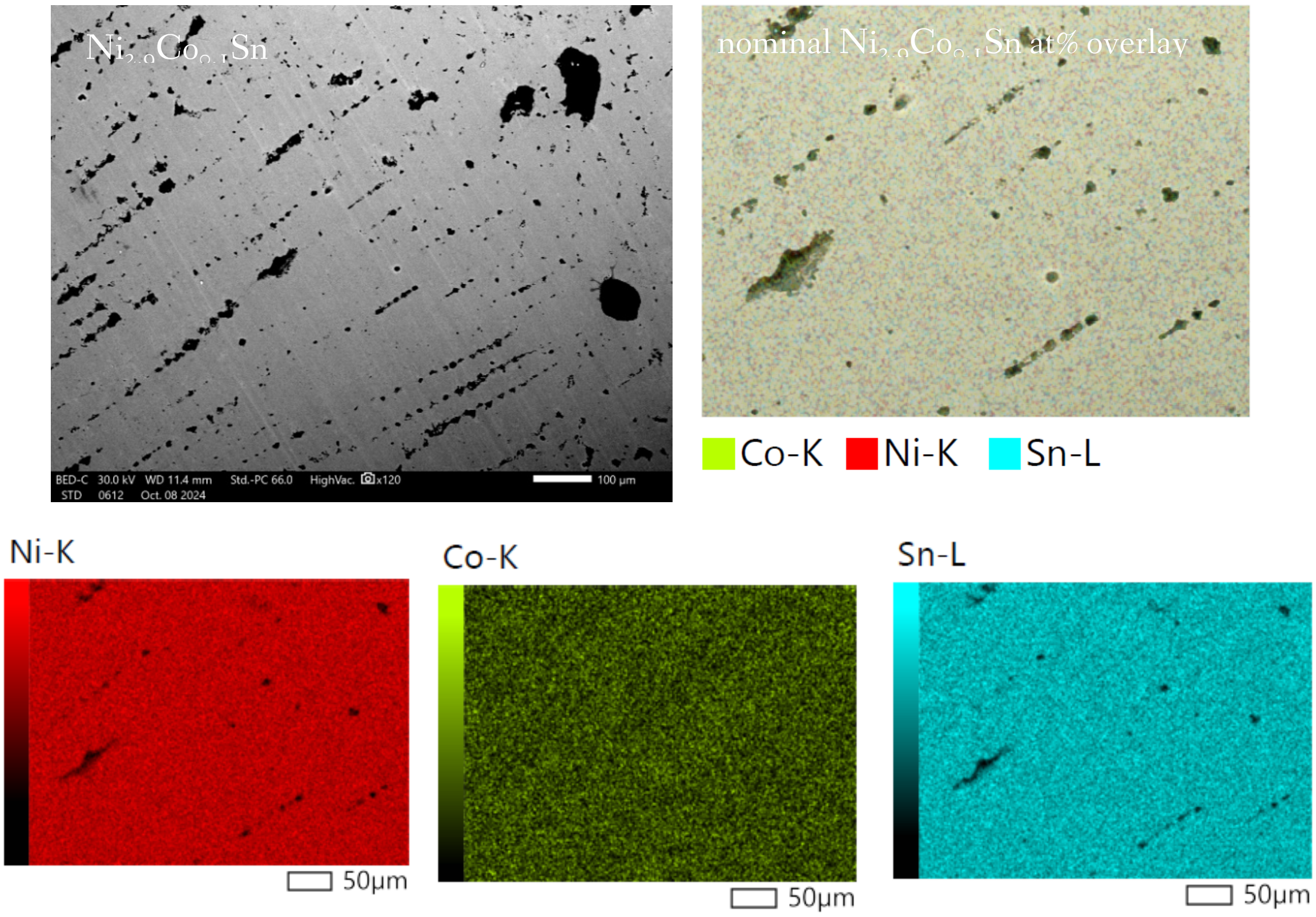

Figure S6: Back scattered detector SEM image, at% composite and individual elemental at% maps for $Ni_{2.9}Co_{0.1}Sn$.

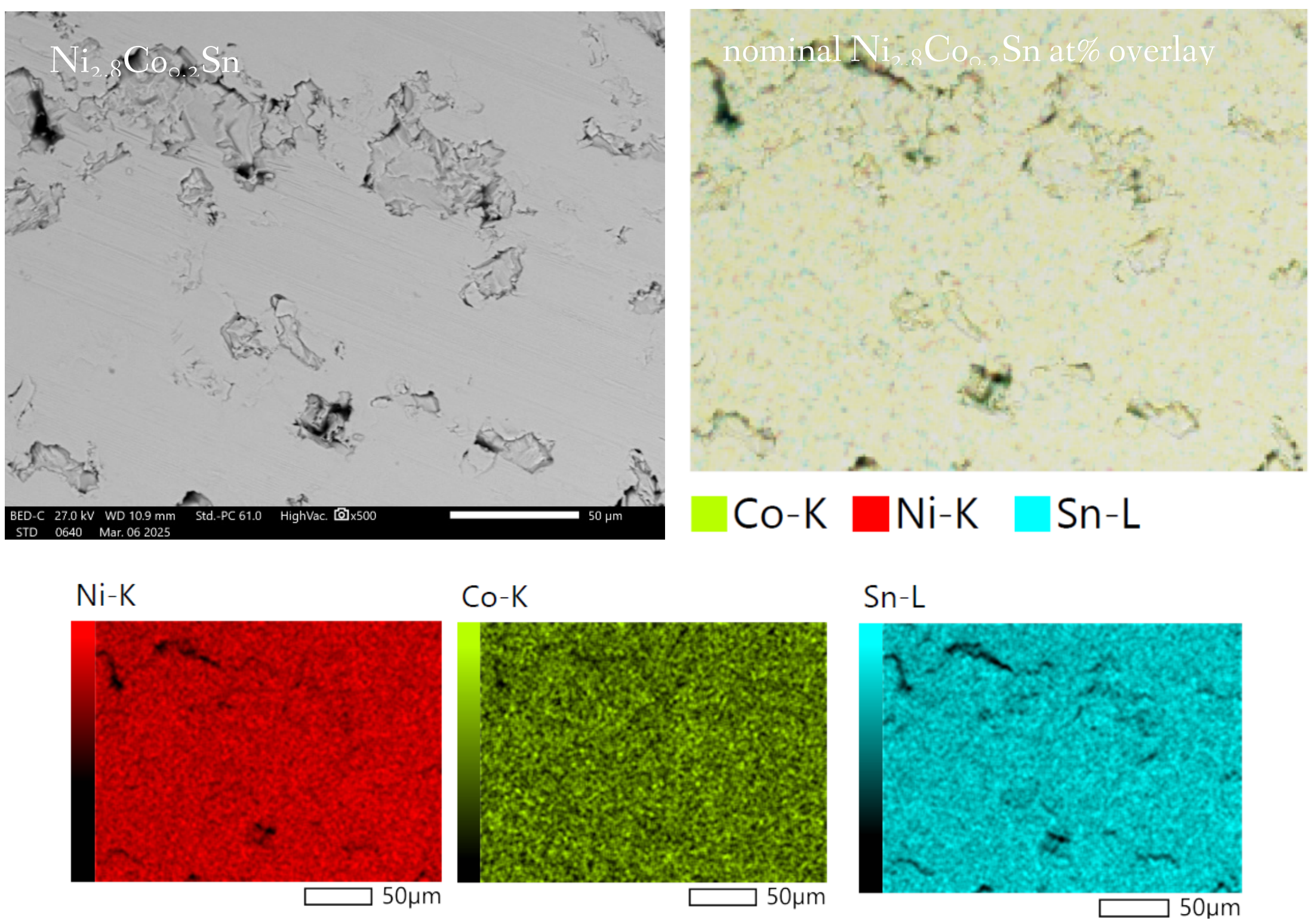

Figure S7: Back scattered detector SEM image, at% composite and individual elemental at% maps for $Ni_{2.8}Co_{0.2}Sn$.

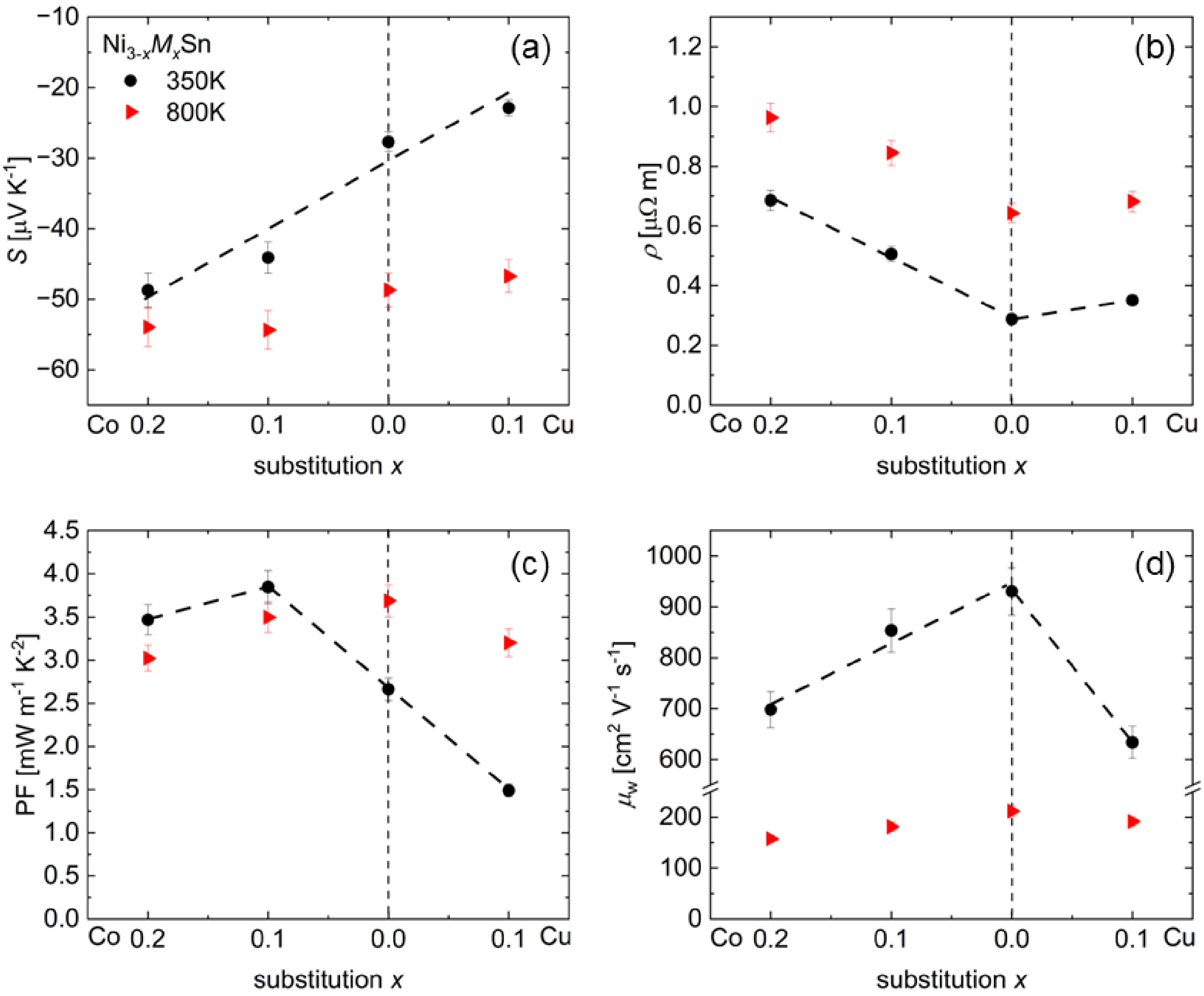

Figure S8: Composition dependence of electric properties of the $Ni_{3-x}M_xSn$ samples at 350 K and 800 K (a) electrical resistivity ρ (b) absolute Seebeck coefficient S (c) Power Factor PF and (d) weighted mobility μ_w.

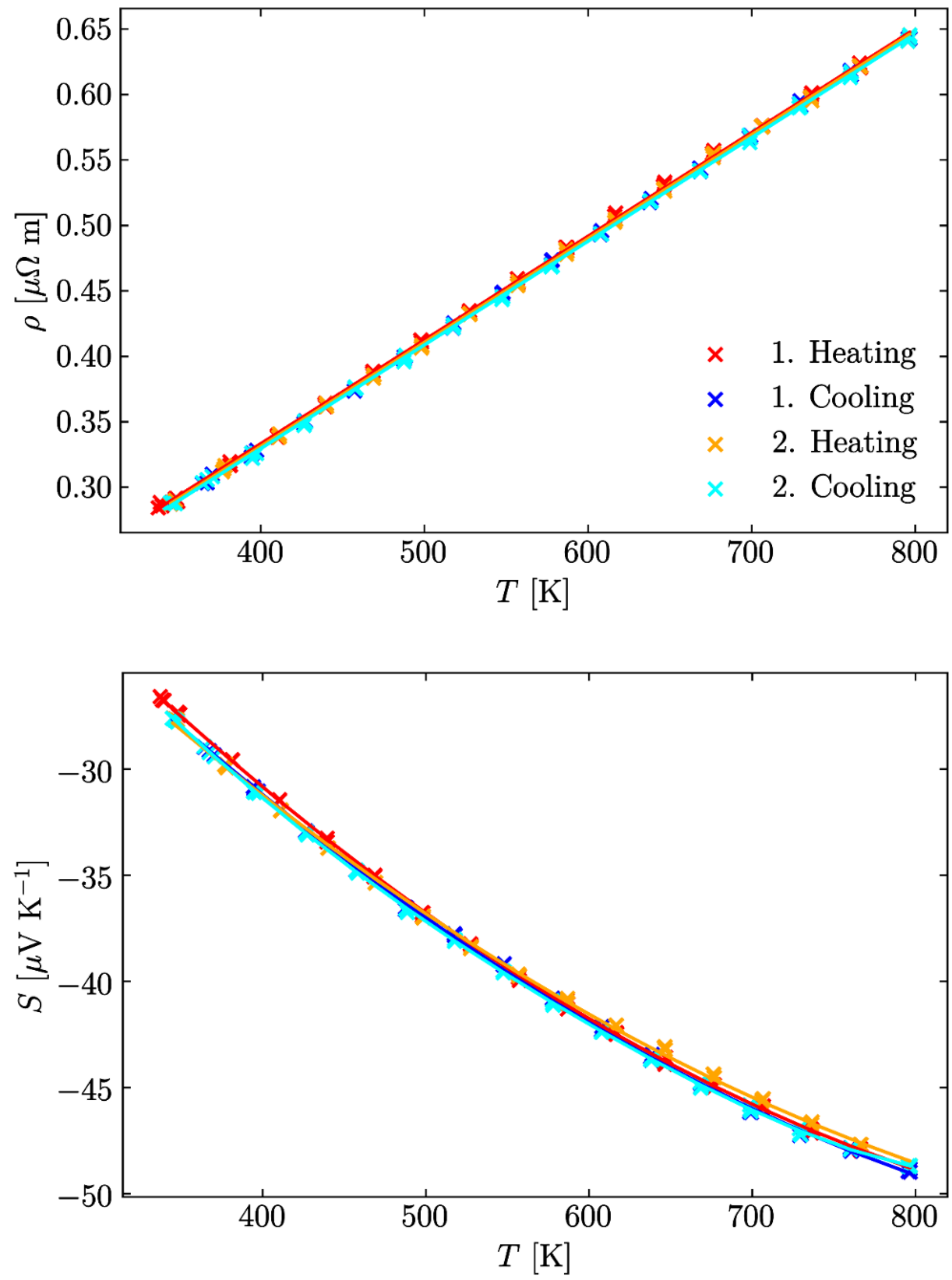

Figure S9: Resistivity and Seebeck measurement of Ni_3Sn. Two complete thermal cycles were measured. Lines are guide to the eye.

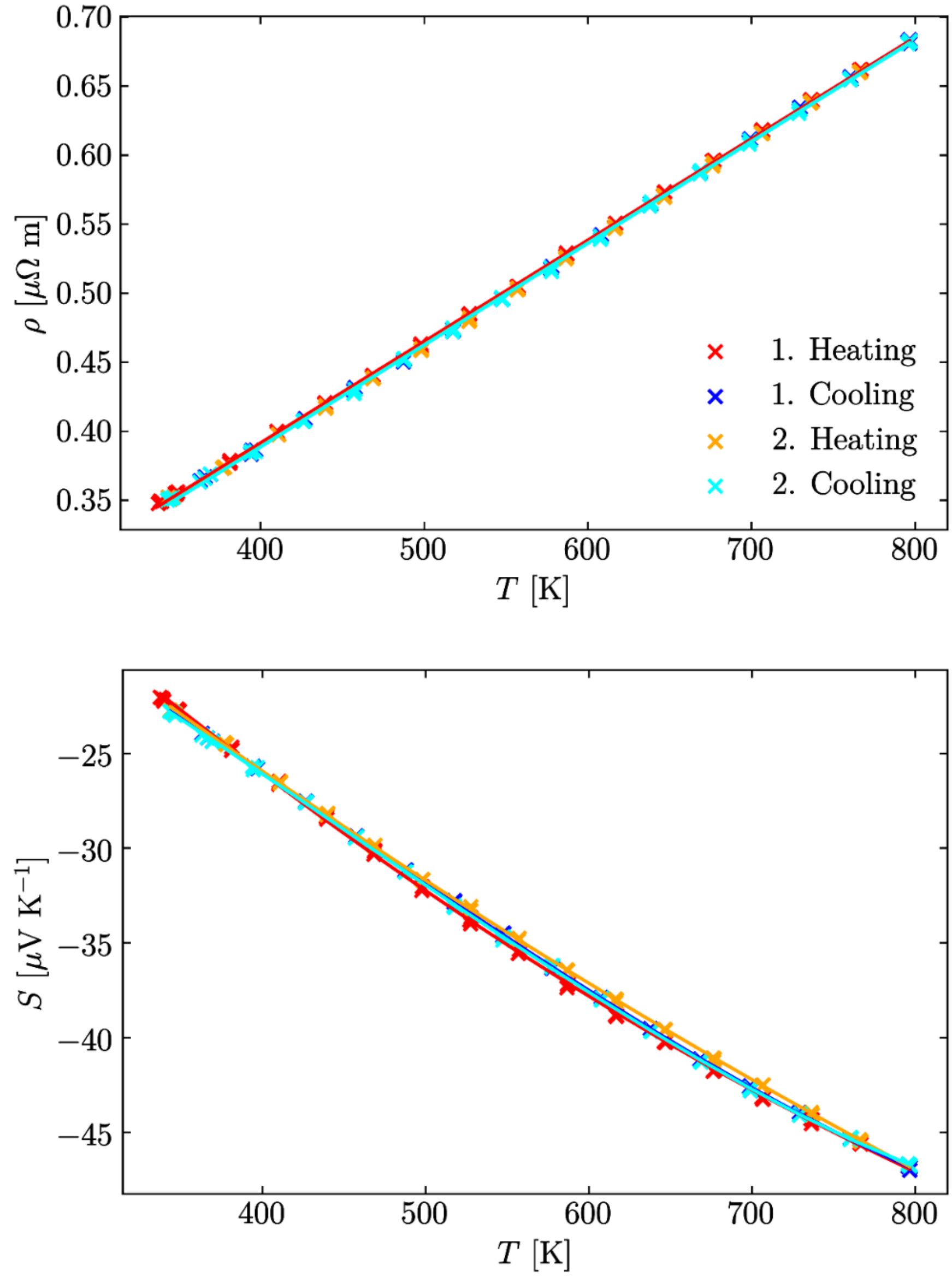

Figure S10: Resistivity and Seebeck measurement of $Ni_{2.9}Cu_{0.1}Sn$. Two complete thermal cycles were measured. Lines are guide to the eye.

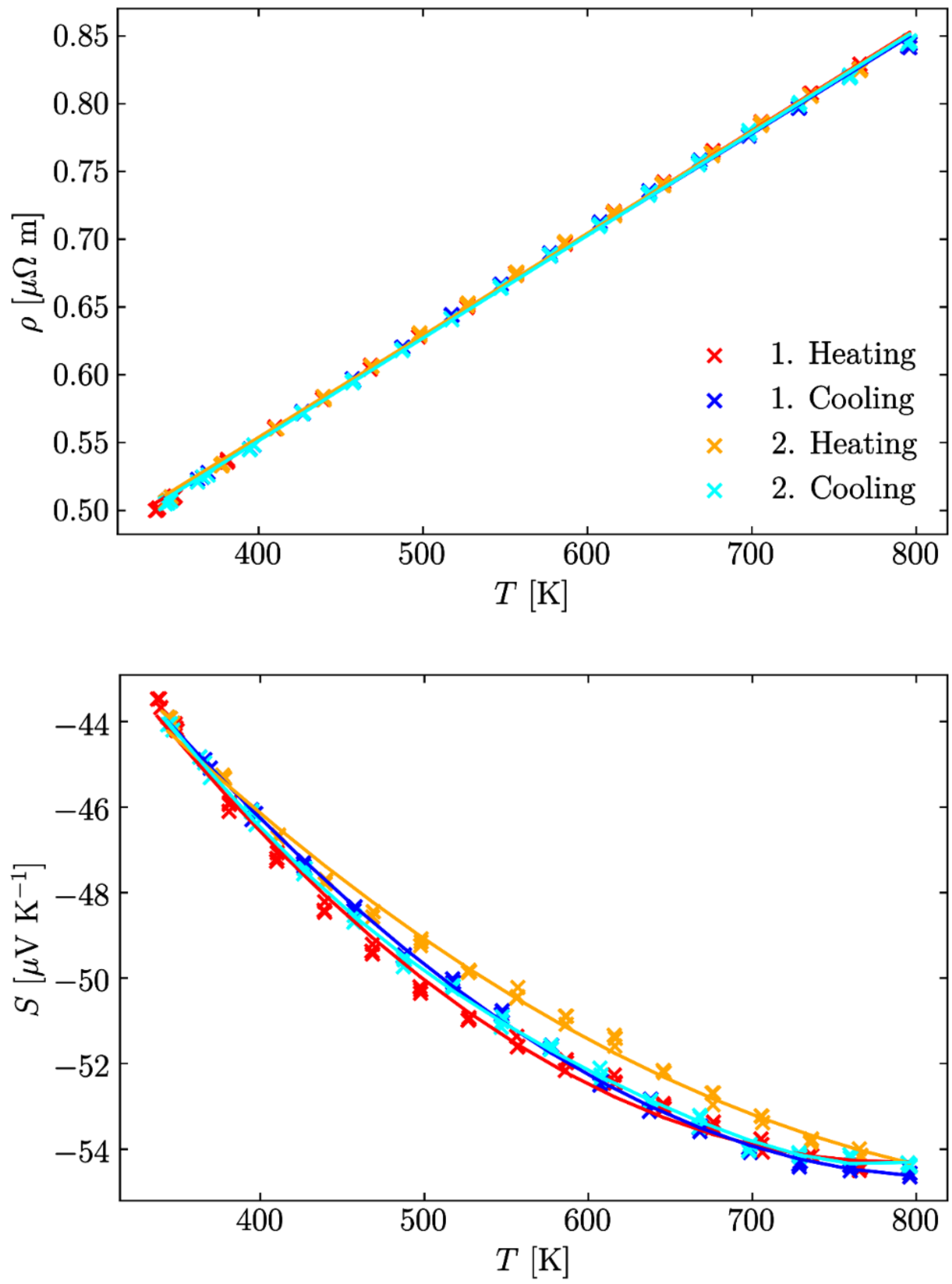

Figure S11: Resistivity and Seebeck measurement of $Ni_{2.9}Co_{0.1}Sn$. Two complete thermal cycles were measured. Lines are guide to the eye.

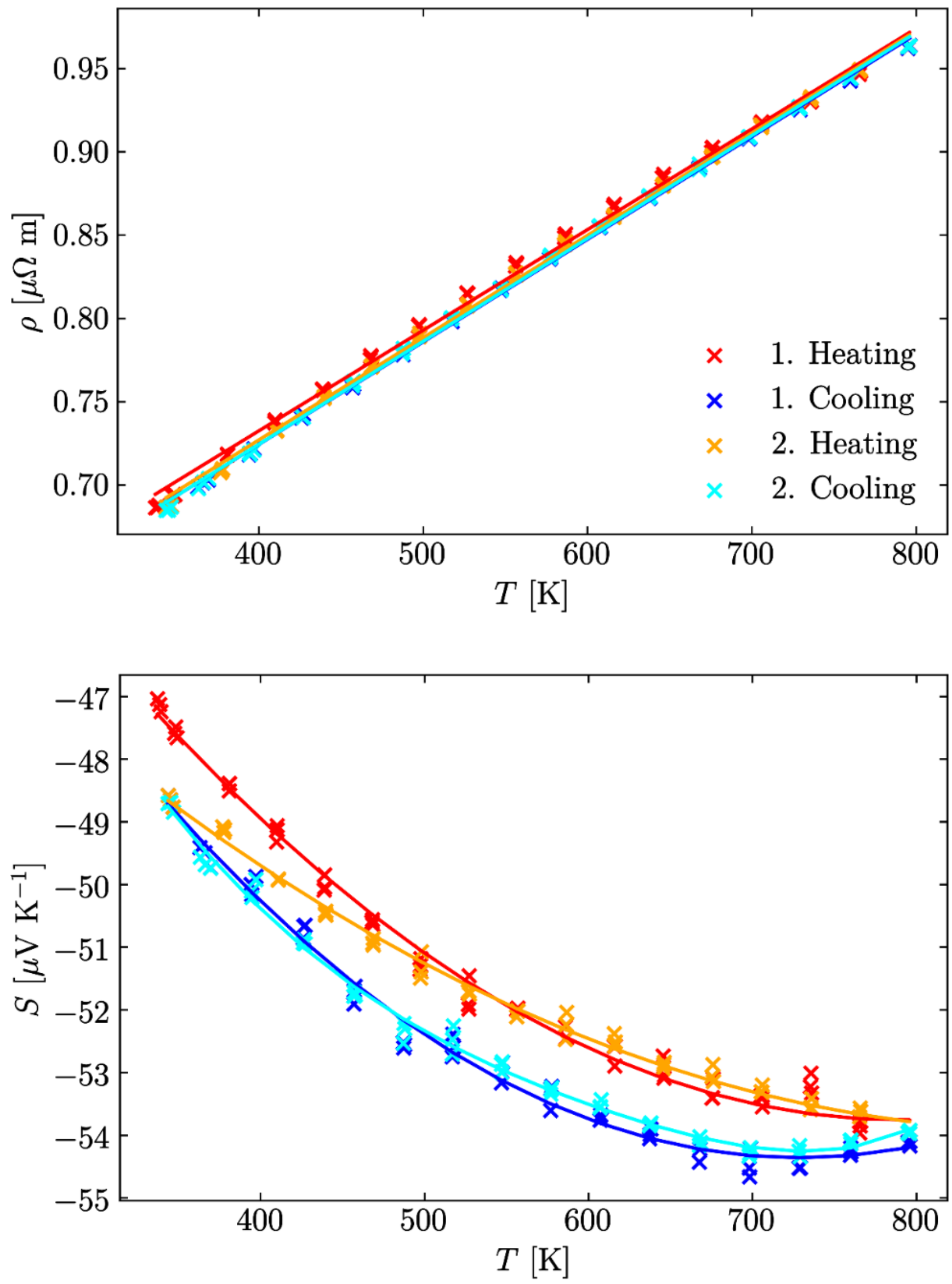

Figure S12: Resistivity and Seebeck measurement of $Ni_{2.8}Co_{0.2}Sn$. Two complete thermal cycles were measured. Lines are guide to the eye.

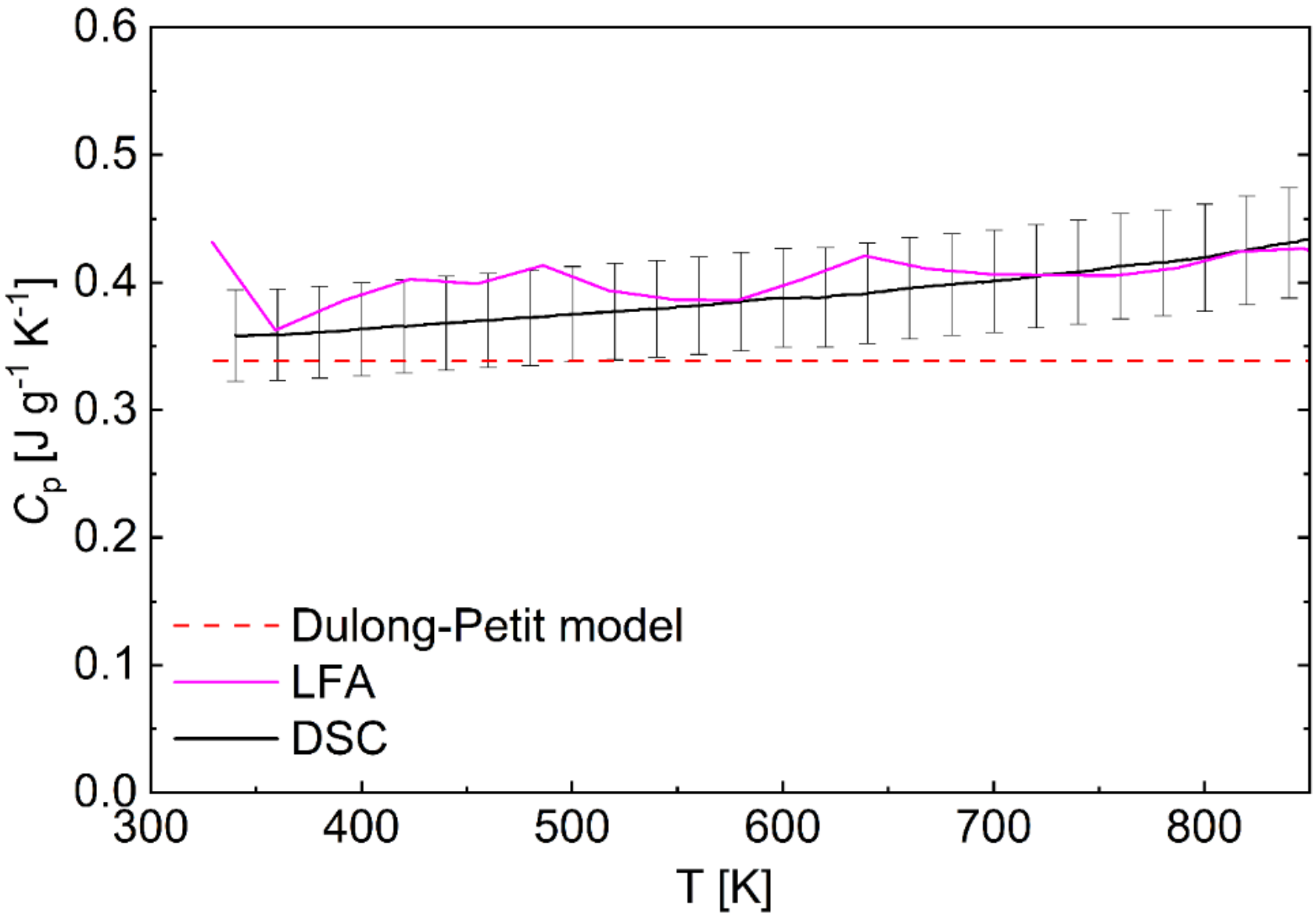

Figure S13: DSC heat capacity (C_P) of Ni_3Sn with data recorded in 1 K steps. The values obtained using the laser flash method (LFA) using a Pyroceram reference and the calculated heat capacity using the Dulong-Petit (DP) model are also shown. This reveals good agreement between DSC and LFA experimental values. The difference with DP is due to electronic and thermal expansion contributions.

II – Supporting DFT and band Modelling

1. DFT based supporting data

a. Partial density of states and flat bands analysis

The projected density of states (PDOS) with GGA-PBE shown in **Figure S14a-c** were computed with consideration of the valence electronic configurations for all relevant atoms: Ni [Ar]3d⁸4s^2 (10 valence electrons), Cu [Ar]3d^{1}⁰4s^1 (11 valence electrons), Co [Ar]3d⁷4s^2 (9 valence electrons), and Sn [Kr]4d^{1}⁰5s^{2}5p^2 (4 valence electrons, counting only 5s , 5p). Within this electron-counting (rigid-band) picture, replacing one Ni with Cu increases the total valence electron count by +1 (electron doping), whereas replacing one Ni with Co decreases it by −1 (hole doping). Across all three systems, Ni-d orbitals strongly dominate the PDOS both below and above the Fermi level, while the second (but smaller) contribution above E_F comes from Sn-p states, hybridized with Ni(d)-Sn(p)[2-4]. **Figures S14d, e** show the flat bands analysis with both element-resolved and orbital-resolved bands, confirming that the Ni-d orbitals are the major contributors both above and below the Fermi level for the overall DOS, but small contributions from Ni-d, Ni-p, and Sn-p are also present above the Fermi level.

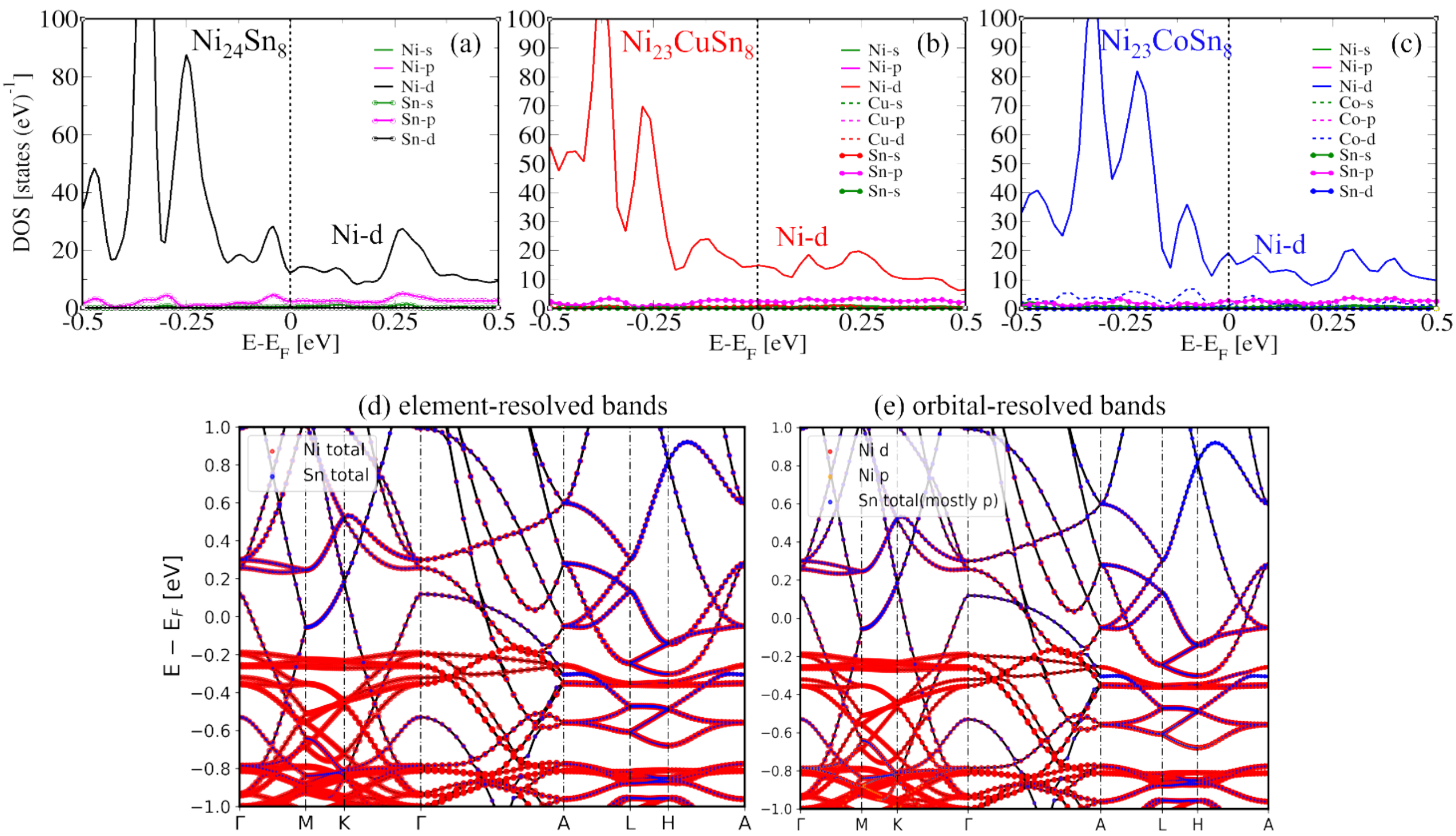

Figure S14: The calculated orbital resolved density of states (DOS) for: (a) the pristine material, (b) the Cu-substituted material, and (c) the Co-substituted material. In all three cases, the Ni-d orbitals' DOS dominates in both sides of the Fermi level. (d) element resolved band structure of $Ni_{24}Sn_8$ showing the total contributions from Ni (red circles) and from Sn (blue circles). (e) Orbital projected bandstructure of $Ni_{24}Sn_8$ showing Ni-d (red circles), Ni-p (orange circles) and Sn (blue circles mostly from p-orbitals) contributions. Flat bands are dominated by Ni-d orbitals below the Fermi level. Above Fermi level, hybridization of Ni-d, Ni-p, and Sn-p orbitals is present, still with Ni-d being the major contributor.

b. Charge density maps and bonding analysis

The evolution of the lattice parameter is consistent with the calculated relaxed structure. The doping percentage in this case is small, which brings some uncertainty in the refinement as well as the relaxed structure parameters. Experimentally, our data shows that both Cu and Co lead to increase in cell volume, although changes are small and at the limit of accuracy of XRD.

We have also investigated the bonding of Co and Cu in the Ni_3Sn matrix. Ni_3Sn is a metallic system, where positively charged metal ions (Ni) are held by the mobile sea of delocalised valence electrons (of Sn). In the Ni_3Sn structure, each Sn is bonded to 12 nearby Ni atoms, 6 in the *ab* plane, 3 above, and 3 below. The bond length in the relaxed pristine structure is 2.643 Å and 2.648 Å, for the Sn-Ni bond in the in-plane and out-of-plane directions respectively (the *ab*-plane). This results in lattice constants of *a* = 5.285, and *c* = 4.235 Å. After Cu substitution at the Ni site, Sn is bonded to both Cu and Ni. The bond length is now 2.691 and 2.714 Å for Sn-Cu in the in-plane and out-of-plane directions, respectively. Such increase in bond length increases the lattice parameter. The increase in bond length is due to the weak attraction of Cu with delocalized electrons, a result of the high electronegativity of Cu compared to Ni. This could be understood from the charge density difference plots, showing charge density minus the superposition of atomic densities (**Fig. S15**). The positive value of Δρ indicates charge accumulation meaning strong bonding with delocalised electrons. **Figure S15** shows the Cu atom having negative charge density ($Cu_{Δρ-}$), which means it has reduced interaction with delocalised charge, as is the case of Sn ($Sn_{Δρ-}$) as well, adding its charge to the delocalised valence electrons.

However, in the case of Co, the difference in the charge density plot indicates accumulation of charge at Co ($Co_{Δρ+}$), more than Ni, which means stronger bonding. In this case, because of increased electron transfer on the Co, the bond length between Sn and Co decreases (2.632 and 2.649 Å). With additional charge transfer to the Co atom, other Sn-Ni bond lengths increase (2.652 and 2.649 Å). So, in both cases the unit cell volume increases, despite their distinct affinity towards valence charge density.

Table S6: Converged lattice parameters and cell volume for $Ni_{24-x}M_xSn_8$ 2x2x1 supercell from GGA-PBE fully relaxed calculations. The values given in parentheses correspond to the primitive cell.

composition	*a* (Å)	*c* (Å)	*V* (Å^3)
$Ni_{24}Sn_8$	10.57 (5.285)	4.24	409.799 (102.45)
$Ni_{23}CuSn_8$	10.60 (5.299)	4.25	413.065 (103.26)
$Ni_{23}CoSn_8$	10.58 (5.289)	4.23	410.384 (102.57)

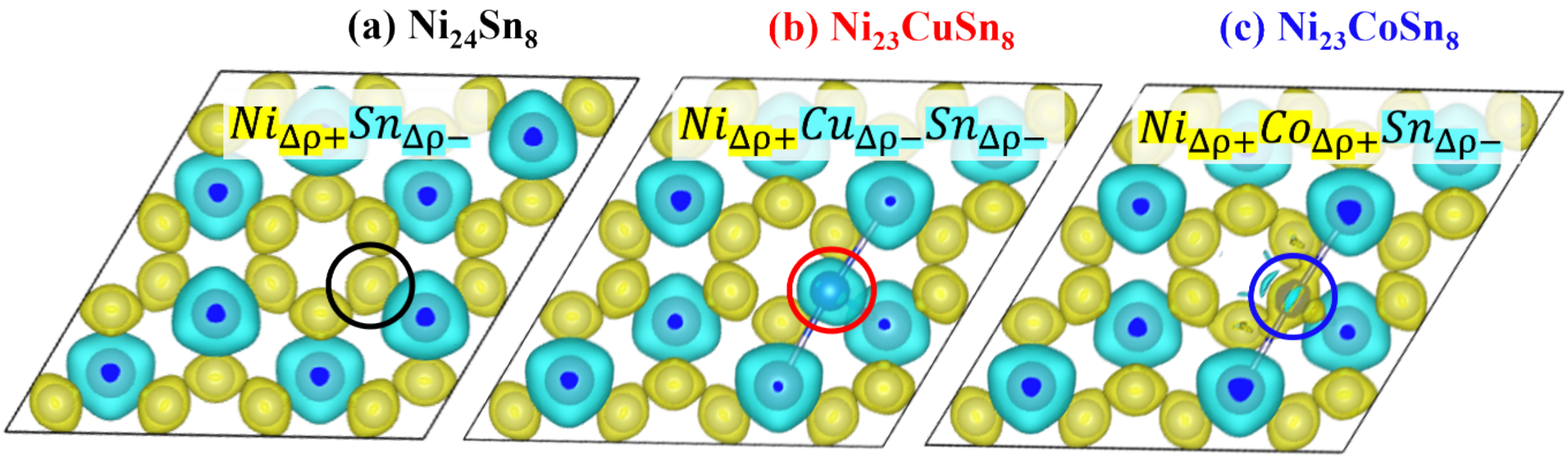

Figure S15: Kagome lattices (ab-plane) with iso-surface plots for the charge density difference (Δρ) between the total and atomic charge densities for: (a) pristine, (b) Cu-doped, and (c) Co-doped systems. The iso-surface regions shown with positive Δρ (yellow color) around Ni and Co, indicate localized electron accumulation, while regions with negative Δρ (zinc color) around Cu and Sn, indicate electron depletion.

c. Two band model development

The total DOS and velocities in the parabolic two-band model are mapped to the DFT DOS and DFT velocities, as shown in **Figure S16** for each system, respectively. These are the quantities used to match the experimental transport data. The hole-like bands' DOS are closer to Fermi level for the Co-substituted system, followed by the pristine material and then the Cu-substituted system. The Cu-substituted alloy has a distinct left-ward shift of -0.028 eV in its DFT DOS relative to the pristine material, but -0.02eV is used in the parabolic model. In contrast, the Co-substituted alloy requires a minor right-ward displacement for the fitted DOS by about +0.04 eV, somewhat larger to the PBE prediction of 0.033eV, but smaller than the GGA+U prediction. The exact deviation in sharp DOS vs best fit in the data is particularly challenging to capture particularly at a fixed Fermi level (used in model as -0.075eV). These shift values are shown in **Figure 4a** (DFT-DOS) and **Figure 5b** (fitting-DOS) in the main paper. Here in **Figures S16a-c**, we explicitly show how the two bands (h-like with the dark blue colour and e-like with green colour) and their total DOS_{fit} in the parabolic model are separately mapped on to the DFT-DOS. Similarly, **Figures S16d-f** show the mapping of DOS-weighted velocities for the model, onto the DOS-weighted DFT velocities separately for each system. A combined plot for all three systems of model velocities is shown in Figure S16 below. Full details behind the development of our model are given in **Section 4.3** in the main paper.

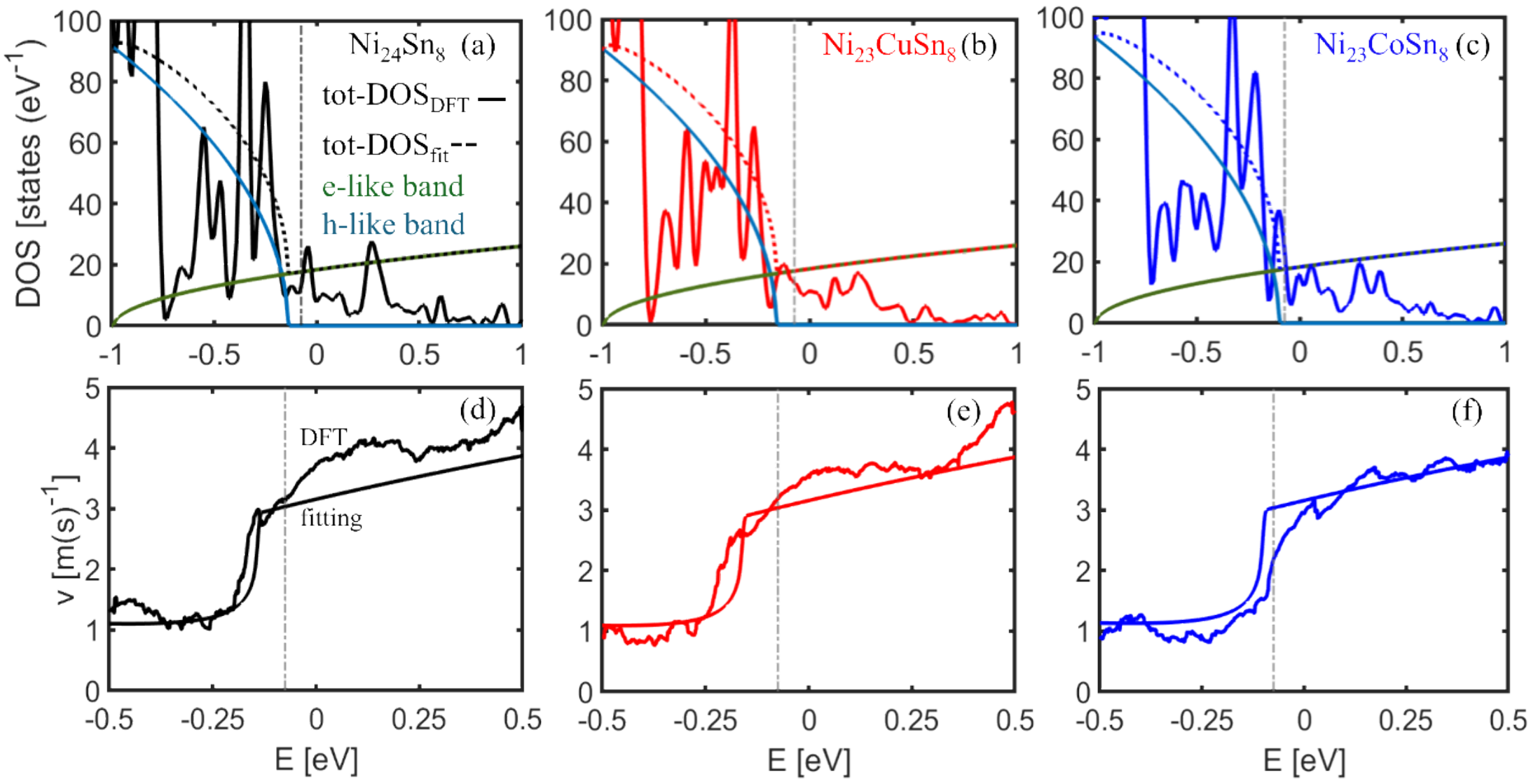

Figure S16: The DFT calculated total DOS (solid lines) and the parabolic fitted total DOS (dotted lines) for the three materials under consideration (labelled) is shown in the upper panel. The total fitting DOS is the sum of the parabolic dispersive/flattened-out bands DOS in the right and left regions, shown individually by the green electron-like band and dark blue heavy hole-like band. Similarly, the lower panel is the total parabolic velocity and total DFT velocity plotted together. The total parabolic DOS and velocity mimic at first order the DFT DOS and velocity in: (a, d) pristine, (b, e) Cu-doped and (c, f) Co-doped systems.

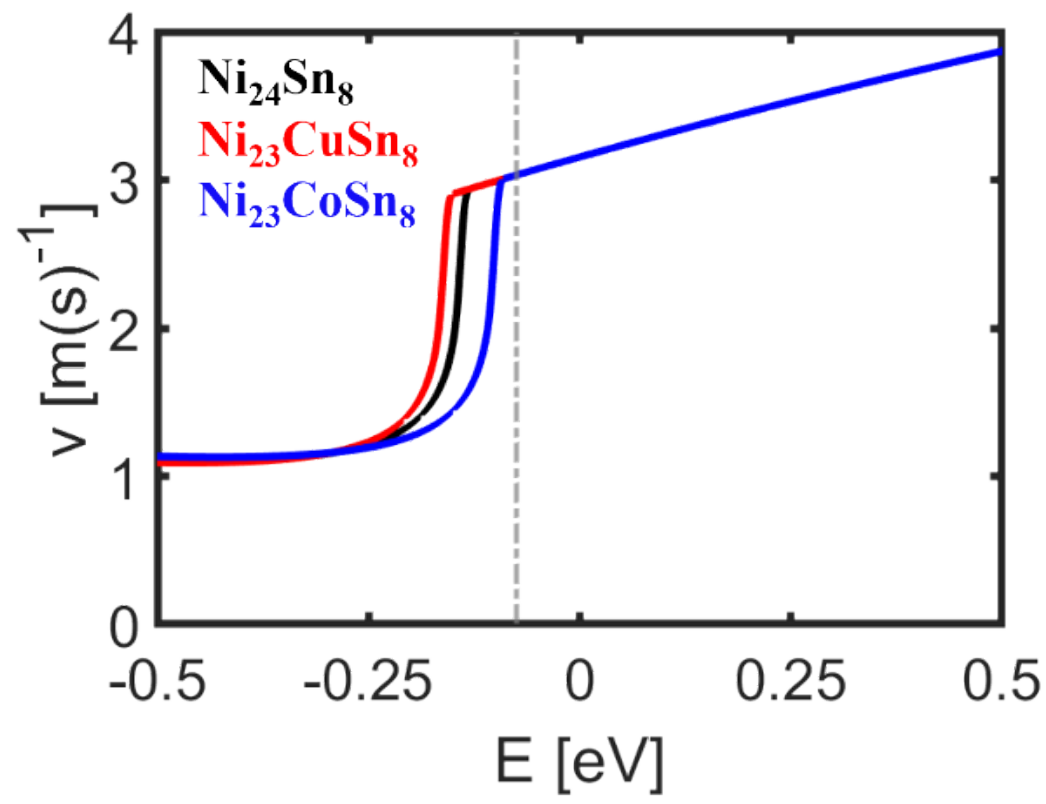

Figure S17: The model velocities plotted using the fitting parabolic two-band model for the three materials.

With these energy dependent quantities (DOS and velocity), the transport distribution function (TDF), calculated at 350 K (initial experimental T value) as a function of energy, is shown below in **Figure S18**, for each system.

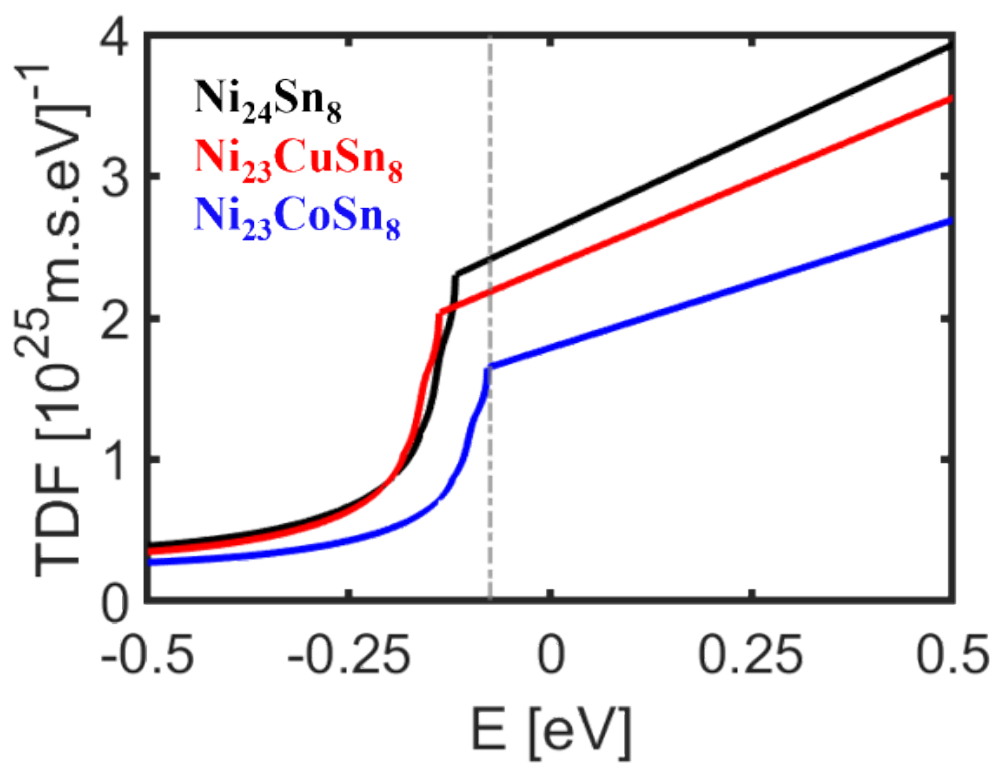

Figure S18: The transport distribution Function (TDF) for the three materials.

The behaviour of *S*(T) using the two-band model with a common set of input parameters including same deformation potentials values (**Table S7**) still captures the main trend as show in **Figure S19**. The parameters used are the averaged parameters of those used in the main paper, except for the position of the heavy-band maxima (sharp DOS rise), which is different for each compound.

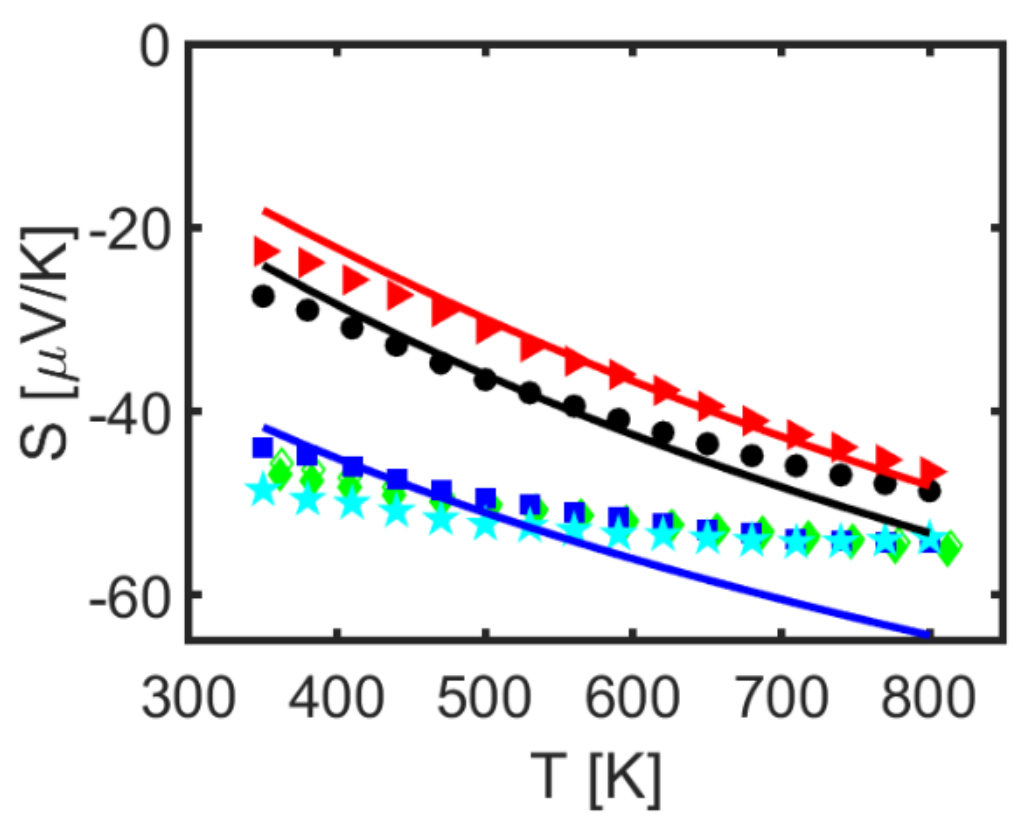

Figure S19: Variation in Seebeck coefficient (solid lines) using common (averaged deformation potentials as well since rest of the parameters are already common in each case) parameters, except for the hole-like band edge. Sample labels are the same as in the main manuscript (red (triangles): $Ni_{2.9}Cu_{0.1}Sn$, black (circles): Ni_3Sn, blue (squares&stars) / green (diamonds): $Ni_{2.9}Co_{0.1}Sn$).

Table S7 shows the parameters used in the parabolic band model to fit the experimental data, for each considered system. This includes the masses of hole- and electron-like bands, the band edges of both bands, and acoustic deformation potential values for both intra- and inter-band scattering mechanisms. The Seebeck coefficient is sensitive to the inter-band deformation potential values which determine

scattering between electron- and hole-like bands, as expected. Increasing the inter-band scattering was an important way to increase the Seebeck coefficient values. In **Table S7**, the band edge of the h-like bands (E_1) is the main parameter responsible for Seebeck coefficient variation.

Table S7: Parameters used to match the parabolic model DOS to the DFT DOS for each system. Here N_v denotes the band degeneracy, 'mass' is the mass of the electron or hole-like bands, and D_{intra} is the intra-band deformation potential, and D_{inter} is the inter-band potential for these bands. E_1 is the band edge of the h-like band, and E_2 is the band edge of e-like band.

composition	h-like mass (N_v)	e-like mass (N_v)	E_1 [eV]	E_2 [eV]	E_F [eV]	$D_{ADP\text{-}intra/inter}$ [eV]	$D_{ODP\text{-}intra/inter}$ [eV]
$Ni_{24}Sn_8$	17 (2)	3.5 (4)	-0.14	-0.99	-0.075	2.80	2.90
$Ni_{23}CuSn_8$	17 (2)	3.5 (4)	-0.16	-0.99	-0.075	3.00	3.00
$Ni_{23}CoSn_8$	17 (2)	3.5 (4)	-0.10	-0.99	-0.075	3.40	3.50
$Ni_{23}CoSn_8$(big)	/	/	/	/	/	3.00	3.20
Average	/	/		/	/	3.05	3.15

The calculated Lorenz number (L) using the two-band model parameters is shown in **Figure S20** and has a weak temperature dependence with value ~2 x10^{-8} W Ω K^{-2}. This is in the middle range between the high density Sommerfeld value and the non-degenerate value (noted by the horizontal lines).

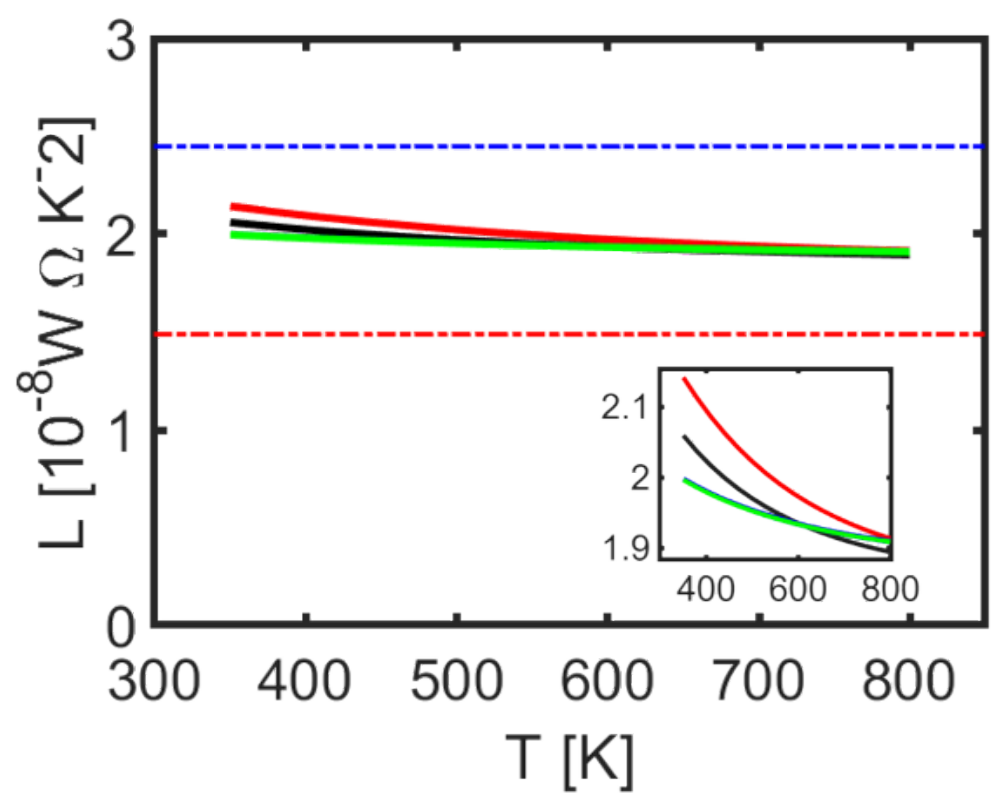

Figure S20: Calculated Lorenz number used to calculate the electronic thermal conductivity for each system. Sample labels are the same as in the main manuscript (red: $Ni_{2.9}Cu_{0.1}Sn$, black: Ni_3Sn, blue/green: $Ni_{2.9}Co_{0.1}Sn$).

Plotted versus Fermi level, as shown in **Figure S21**, all the computed TE properties (conductivity, Seebeck, PF) agree well with experimental data (noted for T = 350 K). These are shown as markers in each plot at the fixed E_F (-0.075 eV) used in the parabolic band model in **Figure 5** of the main paper.

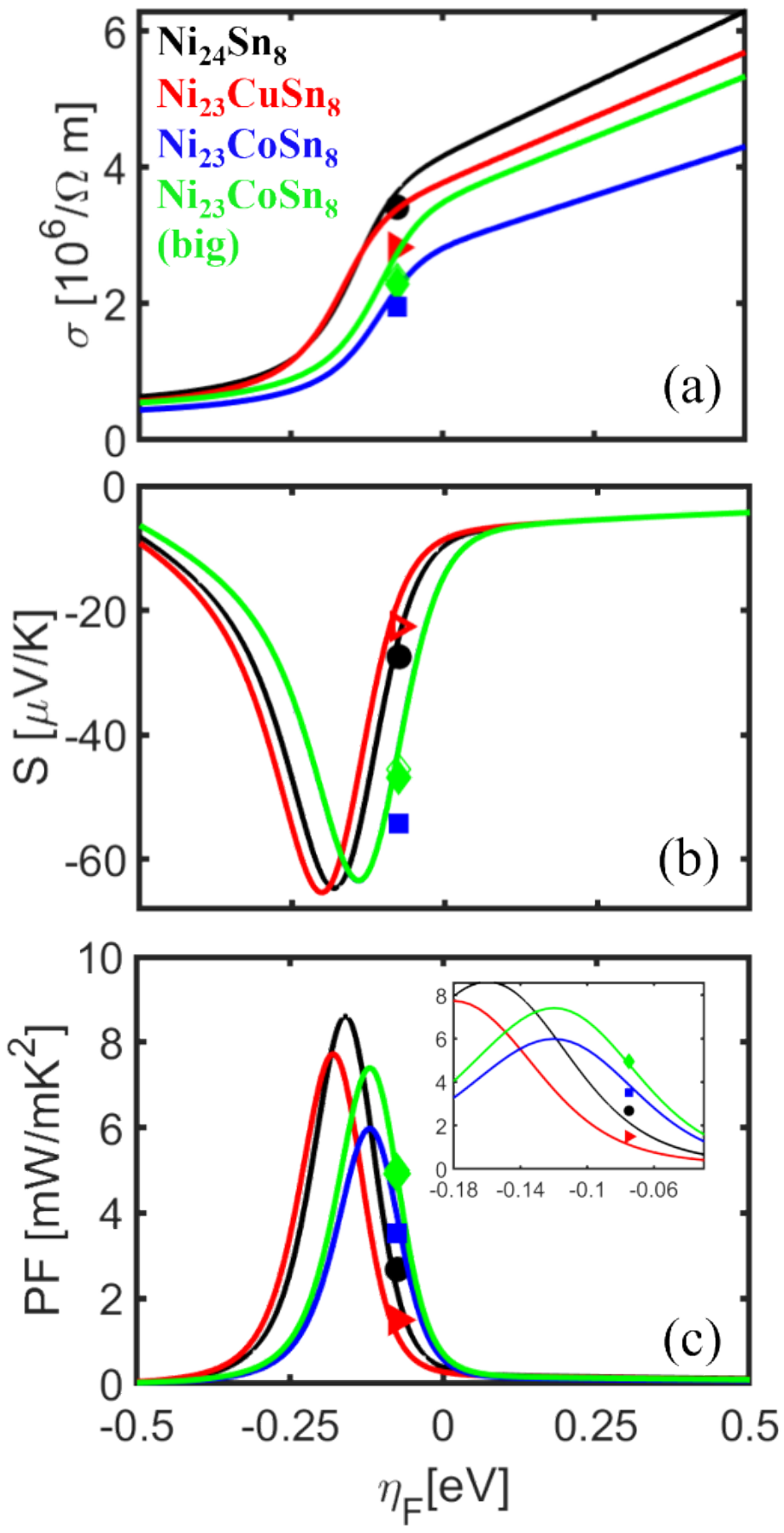

Figure S21: Thermoelectric properties plotted against the reduced Fermi level for all three systems. (a) The electrical conductivity, (b) Seebeck Coefficient, and (c) Power factor with the inset of zoomed PF peaks. For comparison, in each figure the markers show the experimental value for the corresponding system (at the corresponding Fermi level that provides the best fit to experimental data). All calculated coefficients and measured values are plotted at the temperature of $T = 350$ K.

2. DFT vs DFT+U, SOC, and spin-polarized calculations

(a) Effect of GGA and GGA+U on the bandstructures:

We used PBE in the main paper, which has some limitations, thus we compare to GGA+U as well here to fine tune the presence and position of the d-orbitals rise. We are also aware of the limitations of GGA+U, specifically when it comes to the choice of U. Note that HSE06 is computationally prohibitive for the large unit cells we consider (32 atoms). We have considered U= 1 eV to see the relative shift of d-orbital with doping. The bandstructures with both DFT and DFT+U calculations are shown in **Figure S22** below. Minor shifts are observed, but not significant to alter the conclusions. For the DOS itself, we have also calculated the GGA (PBE) electronic structure with better relaxation criteria (see **Fig. S23a**). We then compare to the GGA+U DOS, as shown in **Fig. S23b**. In the case of PBE, the shift between the d-orbitals' raising edge from the pristine case is -0.028 eV and +0.033 eV for Cu and Co, respectively. A smaller peak appears closer to the Fermi level in the Co-case, before the large d-orbital peak as well. This makes the effective shift for Co-material somewhat larger. In the case of GGA+U these change to -0.046 eV and +0.051 eV for Cu and Co, respectively, which are larger than in the PBE approach. However, due to the larger uncertainties in both methods and experiment, we use these values as guidance in the two-band model calculations. The values for the d-orbital shift from the pristine material that we used to match the experimental Seebeck data are -0.02 eV and +0.04 eV (the latter somewhat in between PBE and GGA+U).

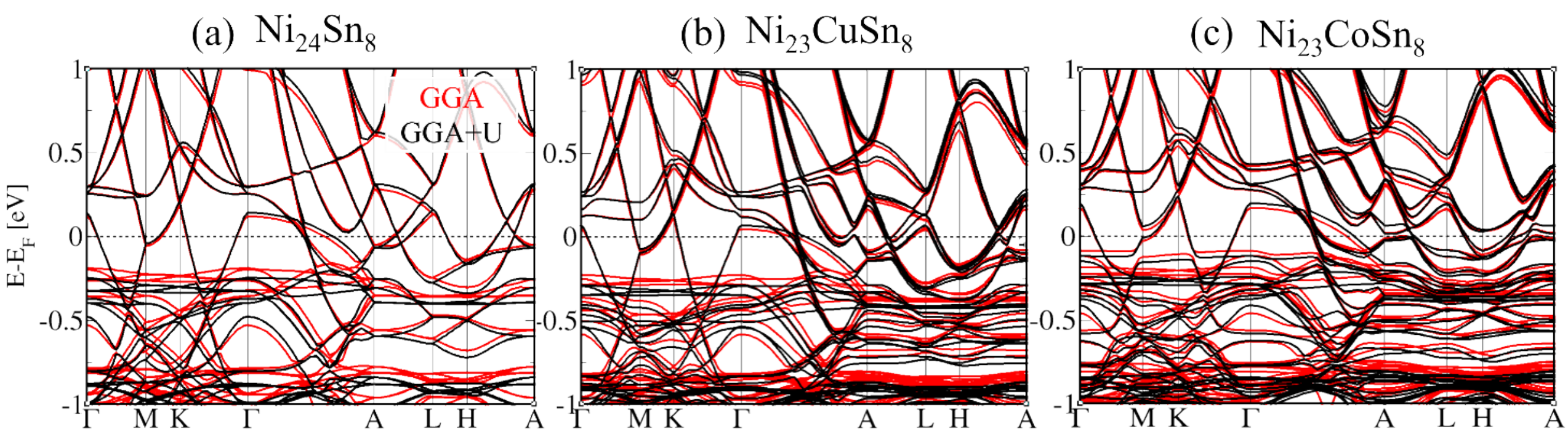

Figure S22: Electronic band structures of: (a) parent material – $Ni_{24}Sn_8$, (b) 4.2 % Cu-substituted compound - $Ni_{23}CuSn_8$ and (c) 4.2 % Co-substituted compound - $Ni_{23}CoSn_8$. Here the red lines in each subfigure are the GGA-PBE calculations, while the black lines are the GGA+U (U= 1 eV) calculations.

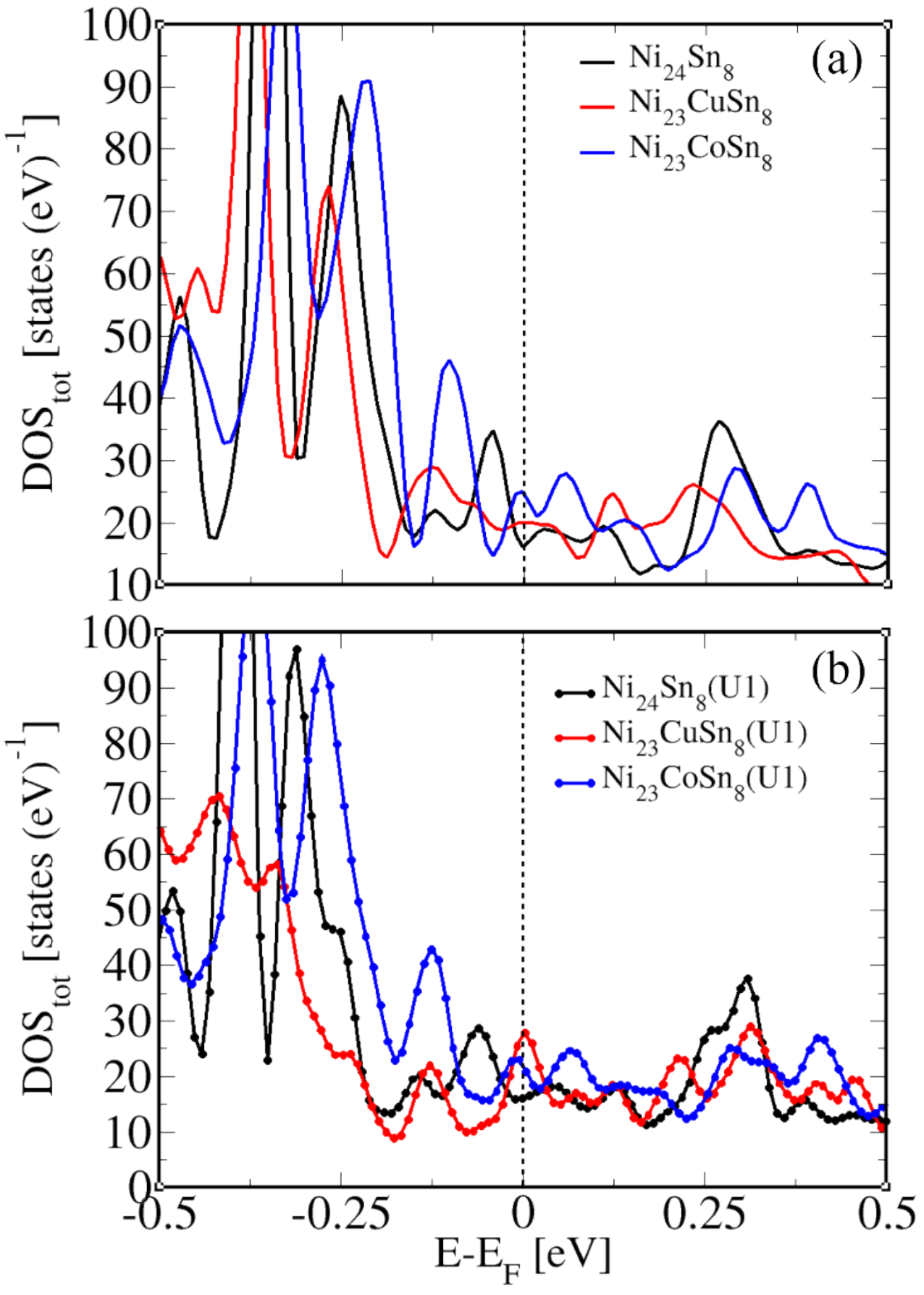

Figure S23: Total density of states of all three systems with: (a) GGA-PBE, and (b) GGA-PBE +U calculations.

(b) Effect of SOC and spin-polarized calculations on the bandstructures:

To examine the influence of spin–orbit coupling (SOC) and spin polarization on the flat bands, calculations were carried out using the pristine Ni_6Sn_2 unit cell rather than the larger supercell, to reduce computational cost. We don't expect significant changes to the SOC for the alloys since we use low Co or Cu doping, thus weak local magnetic moment (the host system is non-magnetic anyway), and we operate at high temperature.

The band structure calculations show that SOC causes only minor band splitting at a few k-points (**Fig. S24a,b** below), with also small corresponding changes in the density of states (DOS) as shown in **Fig. 234** below. Spin polarization produces no apparent changes in the bandstructure (**Fig. 24c**), while the DOS shows that both the spin-up and spin-down contributions are identical with no magnetic order in the system (**Fig. 24d**). Most importantly, the flat band regions remain unchanged in both cases, demonstrating their robustness against SOC and spin-polarization effects.

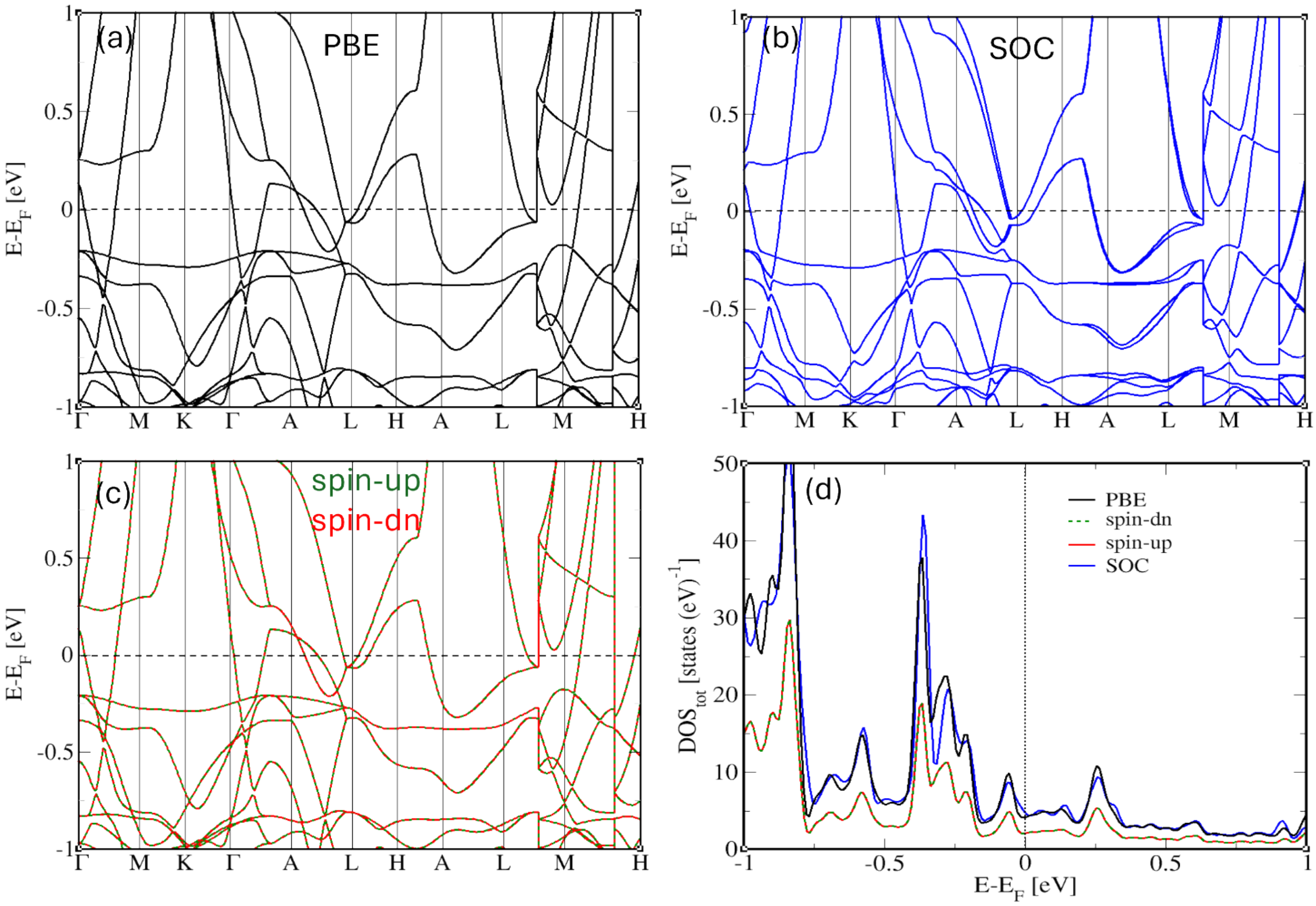

Figure S24: Comparison of the pristine unit cell bandstructure and total DOS for different computational treatment. (a) Bandstructure without spin-orbit-coupling (SOC) and non-spin polarized considerations. (b) Bandstructure including SOC. (c) Spin-polarized bandstructure with spin-up and spin-down bands shown. (d) The total density of states for each bandstructure case.

References

1. F. Garmroudi, J. Coulter, I. Serhiienko, S. Di Cataldo, M. Parzer, A. Riss, M. Grasser, S. Stockinger, S. Khmelevskyi, K. Pryga, B. Wiendlocha, K. Held, T. Mori, E. Bauer, A. Georges and A. Pustogow, *Physical Review X*, 2025, **15**, 15.021054.
2. M. Zhao, J. Blyth, T. Yu, G. L. Causer, H. Zhang, J. Liu, W. Jing, M. T. H. Bhuiyan, Z. T. Liu, M. Ye, Y. Du, Z. Yin, M. S. Fuhrer, A. Tadich and M. T. Edmonds, *Adv Mater*, 2025, DOI: 10.1002/adma.202516003, e16003.
3. X. Bi, X. Hu, Q. Li and Y. Li, *Results in Physics*, 2019, **14**, 102439.
4. A. L. Lyubimtsev, A. I. Baranov, A. Fischer, L. Kloo and B. A. Popovkin, *Journal of Alloys and Compounds*, 2002, **340**, 167-172.
